# The role of MscL amphipathic N terminus indicates a blueprint for bilayer-mediated gating of mechanosensitive channels

**DOI:** 10.1038/ncomms11984

**Published:** 2016-06-22

**Authors:** Navid Bavi, D. Marien Cortes, Charles D. Cox, Paul R. Rohde, Weihong Liu, Joachim W. Deitmer, Omid Bavi, Pavel Strop, Adam P. Hill, Douglas Rees, Ben Corry, Eduardo Perozo, Boris Martinac

**Affiliations:** 1 Division of Molecular Cardiology and Biophysics, Victor Chang Cardiac Research Institute, Darlinghurst, New South Wales 2010, Australia; 2 St Vincent's Clinical School, Faculty of Medicine, University of New South Wales, Darlinghurst, New South Wales 2010, Australia; 3 Department of Biochemistry and Molecular Biology, Institute for Biophysical Dynamics, University of Chicago, Chicago, Illinois 60637, USA; 4 Department of Pharmacology, University of Western Australia, Nedlands, Western Australia 6009, Australia; 5 FB Biologie, University of Kaiserslautern, Kaiserslautern D-67663, Germany; 6 Institute for Nanoscience and Nanotechnology, Sharif University of Technology, Tehran 1458889694, Iran; 7 Division of Chemistry and Chemical Engineering, Howard Hughes Medical Institute, California Institute of Technology, Pasadena, California 91125, USA; 8 Research School of Biology, The Australian National University, Acton, Australian Capital Territory 2601, Australia

## Abstract

The bacterial mechanosensitive channel MscL gates in response to membrane tension as a result of mechanical force transmitted directly to the channel from the lipid bilayer. MscL represents an excellent model system to study the basic biophysical principles of mechanosensory transduction. However, understanding of the essential structural components that transduce bilayer tension into channel gating remains incomplete. Here using multiple experimental and computational approaches, we demonstrate that the amphipathic N-terminal helix of MscL acts as a crucial structural element during tension-induced gating, both stabilizing the closed state and coupling the channel to the membrane. We propose that this may also represent a common principle in the gating cycle of unrelated mechanosensitive ion channels, allowing the coupling of channel conformation to membrane dynamics.

## Abstract (web summary)

The activation of bacterial mechanosensitive channels is still not fully understood. Here, Bavi *et al*. show that the N-terminal helix of MscL dynamically couples membrane tension to channel gating, suggesting a conserved mechanism underlying the mechanosensitivity of ion channels of higher organisms.

## Graphical abstract

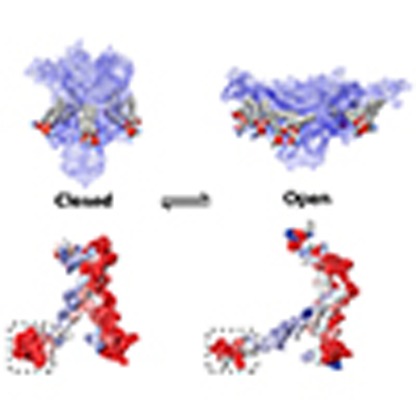

Mechanosensitive channels (MSs) are a ubiquitous type of molecular force sensor^1^,^2^,^3^. They convert the various mechanical forces that regulate and define life at all levels into electrical signals^4^. For this to occur, the applied mechanical force must generate a conformational change that leads to channel gating^5^. Current knowledge suggests that force maybe transmitted via the lipid bilayer as shown for bacterial MS channels, two-pore domain potassium channels and Piezo channels or via tethering of the channel to structural scaffold proteins^6^,^7^,^8^,^9^,^10^. Indeed, MS channels represent a structurally diverse class of proteins, a fact that has largely precluded the identification of a universal ‘force-sensing' motif^11^,^12^,^13^,^14^. Despite this lack of structural similarity much of the knowledge of the basic biophysical principles that govern bilayer-mediated gating of this class of channels comes from studies of the MS channel of large conductance (MscL) from *Escherichia coli* and its homologues^15^,^16^. MscL is a homopentamer, each monomer consisting of two transmembrane (TM) helices: TM1 lines the pore and TM2 interacts with the lipid bilayer and is connected to a coiled-coil C-terminal helical bundle^14^,^17^,^18^. The last structural feature is an amphipathic N-terminal helix, previously named S1 that is connected to the pore-lining TM1 helix via a glycine hinge (G14). During gating and in response to forces transmitted directly from the bilayer, the channel undergoes a large in-plane area expansion^15^,^19^,^20^,^21^,^22^, where the pore-lining TM1 helix tilts and rotates in response to tension, culminating in solvation of a hydrophobic gate^23^,^24^. MscL activation results in the development of a large non-selective pore with a diameter approaching ∼3 nm and a unitary conductance in the range of ∼3 nS (refs 15, 20).

While there is a consensus on most of the major global conformational changes that occur during gating, the critical role of the N-terminal helix in MscL gating cycle remains controversial^19^,^25^. Two competing models have been proposed. The first model suggests that the N-terminal domain acts as a second gate, providing an additional constriction point on top of the hydrophobic lock formed principally by L19 and V23 (refs 24, 26). This model was largely built on the initial MscL crystal structure^14^ that was later refined, particularly concerning the position of the N-terminal helix^17^. The second model suggested by Blount and co-workers^25^ is one in which the N terminus has a close association with the lipid bilayer and acts as a crucial mechanosensing element.

Here using patch-clamp electrophysiology, site-directed spin-labelling electron paramagnetic resonance (EPR) spectroscopy and multiple computational approaches we show that the N-terminal helix of MscL acts as a dynamic membrane-coupling element. In its dual role, the N-terminal helix both associates with the bilayer at the lipid–solvent interface and drives the tilting of the pore-lining TM1 helix, leading to the radial expansion of the pore. The juxtaposition of an amphipathic coupling helix (for example, N terminus) with a pore-lining helix (for example, TM1) through a flexible linker might also be the architectural foundation underlying bilayer force transmission in MscS-like and two-pore domain K^+^ (K_2P_) channels^11^,^27^,^28^,^29^,^30^. This mechanism might also be involved in force transduction for some members of the TRP channel family^31^. Our data suggest that the gating mechanism of MscL, a primordial MS, might reveal a unifying fundamental blueprint that underlies the mechanosensitivity of structurally unrelated ion channels.

## Results

### Conservation of the N-terminal helix

**Figure 1:**
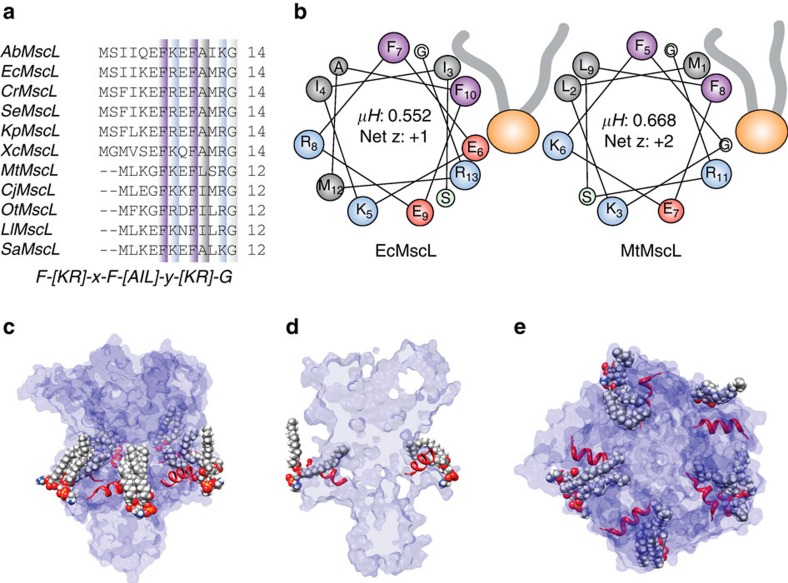
Conservation and amphipathic nature of the N-terminal helix in bacterial homologues of MscL. (**a**) Sequence alignment of MscL homologues from different bacterial classes with the consensus sequence shown below. (**b**) Helical wheel diagrams showing the amphipathic nature of the N-terminal helix of both *E. coli* (EcMscL) and *M. tuberculosis* (MtMscL) MscL. Amphipathic helices classically have high hydrophobic moments (>0.45 μH). (**c**,**d**) This type of helix with a high hydrophobic moment usually interacts at the interfacial region of a lipid bilayer with the hydrophobic face buried. Here the orientation of the N-terminal helix parallel to the membrane plane is shown from an equilibrated MD simulation model of EcMscL (see Fig. 4). Here we can also clearly see how the acyl chains bend over the N-terminal helix and protrude into an inter-subunit cavity. (**e**) Top view of the N-terminal helix showing its orientation and membrane association.

The N-terminal helix of *E. coli* MscL (EcMscL) is widely conserved throughout its homologues. While the absolute length of the helix varies among bacterial species (∼10–14 amino acids), there is a conserved amphipathic region at the distal end of the N-terminal helix, preceding the pore-lining TM1 helix, with the consensus sequence F-[K,R]-x-F-[A,I,L]-x-[K,R]-G (Fig. 1a). These helices have a relatively high % of charged residues (∼20–30 %) indicative of interfacial helices (*c.f.* 5–10 % in TM helices). In addition, all these N-terminal helices have a net positive charge with the exception of AbMscL, as one would expect from an intracellular helix, in agreement with the positive inside rule. These helices also have a high hydrophobic moment (<*μH>*), again a characteristic of interfacial amphipathic helices (Fig. 1b). Using the consensus sequence shown in Fig. 1a as a search motif identifies a number of proteins known to associate and bind to membranes, for example, phycobiliprotein ApcE, C2 domain containing proteins and bacterial glucosyltransferase enzymes.

This interfacial positioning is supported by our equilibrated molecular dynamics (MD) simulations, which are discussed fully later in the text (Fig. 1c–e).

### Interfacial positioning and dynamics of the N-terminal helix

Although the N-terminal segment could not be modelled in the original crystallographic analysis of the *Mycobacterium tuberculosis* MscL (MtMscL), a subsequent re-refinement of the original diffraction data revealed ordered density for this region^14^,^17^. MscL crystals were obtained from a construct with an additional 23 residues at the N terminus, including a decahistidine tag, and removal of this tag was not necessary for crystal growth. These residues are presumably disordered in the crystal structure.

To probe both the interfacial nature of the N terminus and the influence of the N-terminal tag on structure and dynamics, we generated three different constructs (Supplementary Fig. 1a) with histidine tags at the N- and C termini, plus an additional construct with a deleted C-terminal helical bundle (Δ110). Individual cysteine mutants were introduced in each construct background at positions 2–12. In all cases, mutants were purified as detergent-stabilized (*n*-Dodecyl β-D-maltoside) pentamers and were stable at room temperature, as has been the case in previous studies for the majority of cysteine mutants of EcMscL^15^,^32^. This is taken as an indication that, overall, cysteine mutagenesis does not have major consequences for the structural integrity of this region of the channel molecule. However, this is not to suggest that these mutations are functionally irrelevant (Supplementary Fig. 1b). Most mutations in the N terminus showed an increased pressure threshold and many displayed frequent subconducting states, with one of the most severe in our hands being K5C. In order to show that, despite this loss of sensitivity, the channel could still adopt the open state we compared K5C with the wild-type (WT)-like mutant I24C (Supplementary Fig. 2). The K5C mutant required more lysophosphatidylcholine (LPC) to gate in EPR experiments but ultimately reached the fully open state. This suggests that under conditions that favour ion flux, attaching spin labels in the N terminus does not preclude the channel from making the closed to open transition. We also confirmed the spin-labelling efficiency at these sites using mass spectrometry (Supplementary Fig. 3).

Initial examination of the EPR spectra derived from the spin-labelled N terminus mutants (Supplementary Fig. 1a) reveals that the overall dynamics of this region increases considerably when the His-tag peptide is covalently attached. Changes in individual probe mobility were assessed from line shape differences (the inverse of width in the central resonant line, 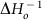). In particular, for the N-terminal His-tag constructs spin labels at positions 2–6 show mobility parameter values typically associated with flexible loops or disordered regions (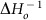
>0.3). When the His-tag peptide was attached to the C terminus, of either the full length or truncated channel, local dynamics were periodic, suggestive of a better-defined secondary structure.

**Figure 2:**
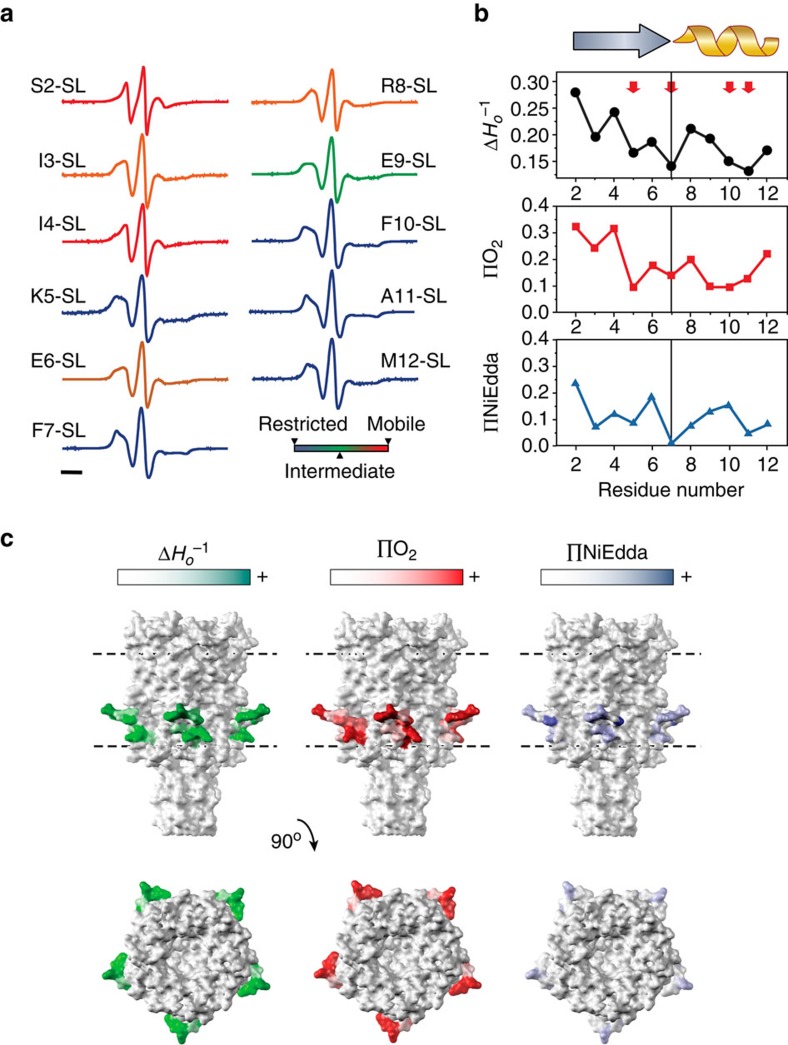
Site-directed spin-labelling analysis of the MscL N terminus. (**a**) X-band CW-EPR spectra of spin-labelled N-terminal mutants reconstituted into azolectin liposomes. Spectra are colour-coded according to their overall probe dynamics as shown in the colour gradient (bottom right). (**b**) Environmental parameter profiles derived from the spectra in **a** or from power saturation experiments. Mobility parameter Δ*H_o_*^−1^ (top panel, black circles), oxygen-accessibility parameter ΔO_2_ (centre panel, red squares) and NiEdda-accessibility parameter Δ_NiEdda_ (bottom panel, blue triangles). A cartoon of the assigned secondary structure for this segment is shown on top. Bar represents 20 G. (**c**) EPR-derived structural data mapped on MtMscL. The side and top (extracellular) views of the mapped EPR data are displayed on solvent-accessible surfaces calculated in UCSF Chimera with a probe radius of 1.4 Å. Green tones, probe mobility (Δ*H_o_*^−1^). Red tones, oxygen-accessibility parameter (ΔO_2_). Blue tones, NiEdda-accessibility parameter (ΔNiEdda). The dotted parallel lines represent the putative location of the lipid bilayer.

We then looked specifically at the lipid and water accessibility of the N-terminal helix using our C-terminally linked His-tag construct. Figure 2a shows spectra from residues 2 to 12 of the MscL N-terminal region. The probe mobility (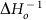) at each of these positions in the resting state is shown in the upper panel of Fig. 2b. These values are matched to the individual spectra shown in Fig. 2a to visualize the degree of mobility of each residue. In particular, the mobility parameter is high prior to residue E6, suggesting that even without an N-terminal His-tag the most proximal end of the N terminus is not helical. Of note there are a number of residues that are particularly restricted, including K5, F7 and F10 (Fig. 2b, upper panel). There is also an increase in the periodicity beginning at F7, indicative of a helical structure, which matches well with our consensus sequence for the bacterial N-terminal helices (Fig. 1a). Figure 2b also indicates the degree of membrane lipid accessibility (O_2_ collision frequency, ΠO_2_) and accessibility to the aqueous environment (NiEdda collision frequency, ΠNiEdda) of residues 2–12 of the N-terminal domain using power saturation experiments^15^,^33^. When we map the degree of accessibility of all these residues on to the crystal structure of MscL, we observe not only the interfacial positioning of the N-terminal domain but also a lipid-accessible inter-subunit cavity (Fig. 2c). As shown in Fig. 1c–e, this is also supported by our MD simulations, demonstrating that lipid acyl chains protrude into these regions in a similar way to that suggested for MscS^34^.

### Global rearrangement of the N-terminal helix

**Figure 3:**
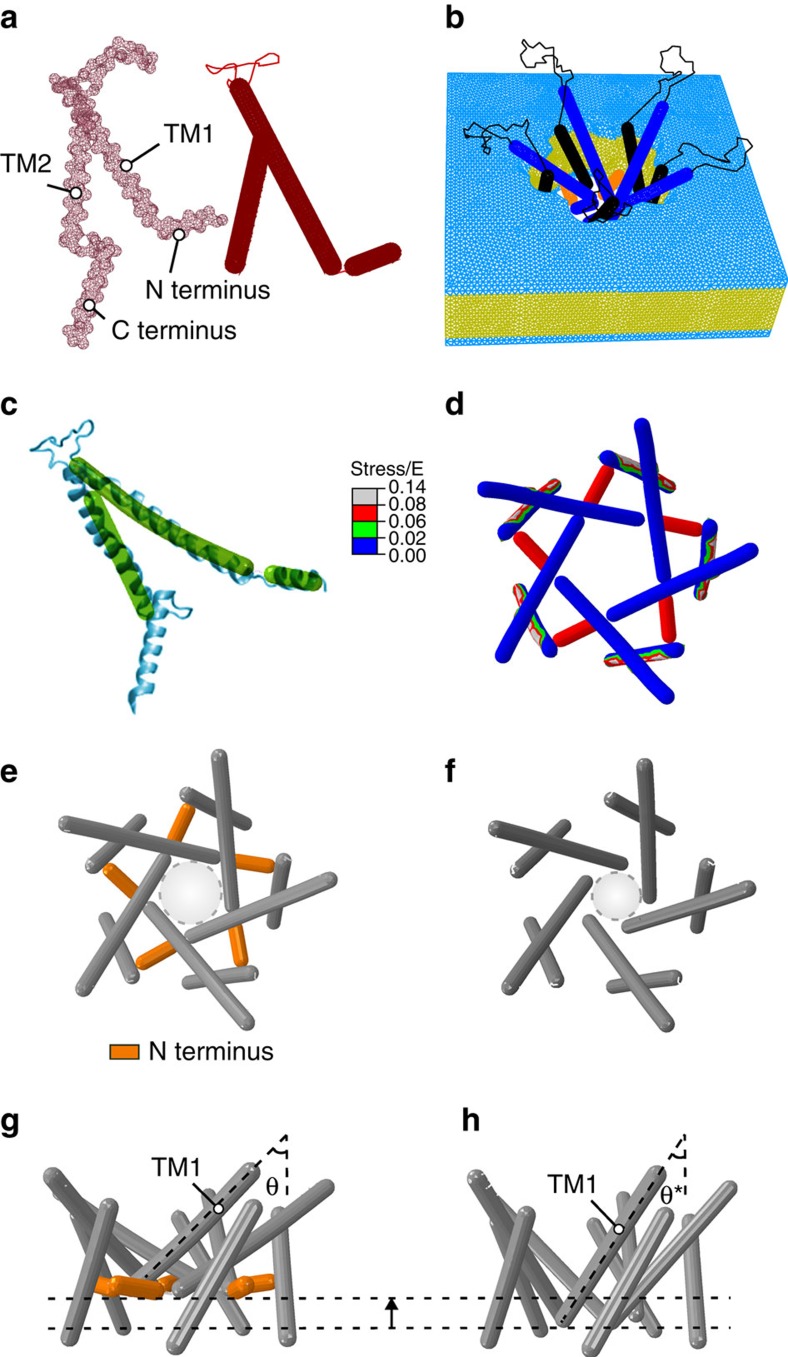
The crucial role of the N-terminal domain in the gating of MscL shown by a parametric FE simulation. (**a**) Mesh representation of a subunit of WT EcMscL obtained from an EcMscL homology model based on MtMscL (PDB: 2OAR; left panel—red mesh)^17^ and the FE model of a subunit of EcMscL without the C-terminal domain (right panel—solid red rods). The α-helices are modelled as elastic rods and the loops are modelled as nonlinear springs. (**b**) The FE structure of EcMscL is embedded into the lipid bilayer with the mesh distribution shown. (**c**) Superposition of FE EcMscL open structure with a previously obtained restrained MD simulation of EcMscL^20^. (**d**) Effective (Von Mises) stress distribution in the open state (top view). The membrane tensional stress is made dimensionless using the Young's modulus of EcMscL, *E*, that is, stress/*E*. The nondimensional stress is 0.6. (**e**,**f**) Channel pore in an expanded state, with and without the N terminus (top view). The nondimensional exerted stress on the membrane is 0.3 in both models, and thus they do not represent fully open structures^20^,^35^. The light grey dashed circles in **e**,**f** represent the position of the effective pore with respect to the plane of the membrane. This diameter is, however, not the actual pore size, since it does not show the side chains on each TM1. The effective pore diameter of the WT model is ∼24 Å, and the model that lacks the N terminus is ∼18 Å. (**g**) Side view of a WT subunit showing that the angle between the N-terminal domain and the TM1 helix increases as the channel begins to gate. Moreover, they both tilt upwards towards the membrane midplane as the membrane is stretched. (**h**) TM1 has less out-of-plane tilting in the absence of the N terminus (θ*=33°) compared with the WT channel (*θ*=45°). Overall, these results suggest that the N-terminal helices have a significant role in transferring the force from the lipid to the pore-lining TM1 helix, guiding both its tilting and expansion.

As a proof of principle for the dynamic role of the N-terminal domain in the gating of MscL, we created a novel finite element model. Finite element (FE) simulations lack the atomistic resolution and information provided by MD including solvation effects, but the advantage of FE simulations being computationally inexpensive means that larger timescales can be probed. The FE models of MscL presented in this study were developed to provide a structural framework for a mechanistic understanding of the gating mechanism of MS channels at the continuum level (Fig. 3a,b). The FE model displays many of the attributes and features of MscL channel gating, reflecting well the pore expansion, TM tilting and their movement away from the central fivefold axis of the channel that is suggested in other studies (Fig. 3c,d)^15^,^19^,^20^,^21^,^22^,^35^,^36^,^37^. Moreover, in the open state, the lipid bilayer thins ∼15% (∼5 Å), which is in good agreement with previously reported results^38^,^39^. In response to membrane tension, the TM1 and TM2 helices move together in an outward radial direction tilting towards the plane of the membrane. The helical axis for TM1 tilted by 21° with respect to the central fivefold axis, whereas the TM2 helices tilted by more than 19°. One important point to note is that the N-terminal helix begins to align with TM1 as a contiguous helix in the open state (Fig. 3c). In addition, a stress analysis illustrates a high level of stress in the N-terminal helix, which points towards it being an important structural mechanosensing entity (Fig. 3d).

When we removed the N terminus, the global rearrangements of the channel were very different under the application of membrane tension (Fig. 3e–h). The tilt of TM1 was not as pronounced and the effective pore radius under the same applied force was almost 50% smaller. The model lacking the N terminus also displayed a lack of stability. It is important to note that, because of the instability of the model without the N terminus, the MscL structures shown in Fig. 3e–h were modelled at half of the nondimensional membrane tension required for the full opening. Thus, the structures shown do not represent fully open states of the channel. Moreover, the ‘effective' pore only takes into account the backbone of the helices. This is because of the fact that both TM1 and TM2 are modelled as elastic rods with a diameter of 5 Å. Therefore, what we define as the effective pore does not represent the exact diameter of the hydrophobic constriction point of the channel. Despite this, the FE model provides us with insights that can be further probed using EPR, MD and mutational analysis.

### Probing the effect of N-terminal deletions

**Figure 4:**
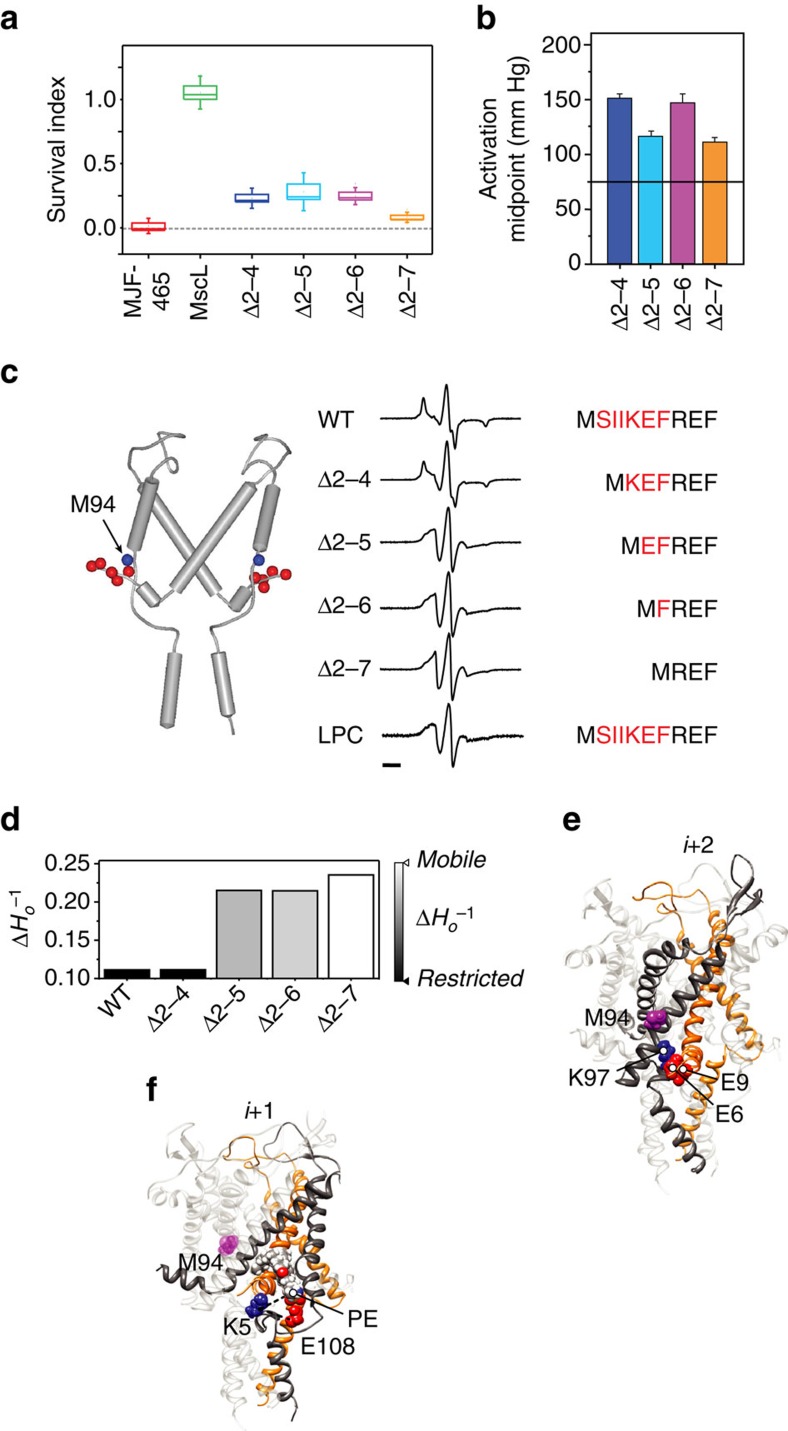
Probing the functional role of the N-terminal domain and the physical interaction between the N terminus and TM2 by deletion analysis. (**a**) Survival of MJF465 (MscL^−^ MscS^−^ MscK^−^) *E. coli* expressing different deletion constructs of MscL after downshock from LB supplemented with 500 mM NaCl to LB (∼1,000 mOsm). Data shown as box plots indicating the mean, 25 and 75 percentile (box) and ±s.d. (capped lines). (**b**) Midpoint of pressure activation of individual deletion constructs determined from multichannel patches (*n*=4, Δ2–7, *n*=3; mean±s.d.). The dotted horizontal line represents the mean value for WT MscL. (**c**) Local dynamics at the intracellular end of TM2 (spin-labelled at position M94) as a function of N-terminal deletions. Left, cartoon representation of subunits *i* and *i*+2 showing the position of the spin-labelling site (blue sphere) and the individual residues in the N terminus that were deleted (red spheres). Right, EPR spectra of M94-SL in a WT background, four different deletions (Δ2–4, Δ2–5, Δ2–6 and Δ2–7) and the WT background opened in the presence of LPCs. The N-terminal sequence of MscL is shown with the region of deleted residues in red. Bar represents 20 G. (**d**) Calculated mobility parameter at position M94-SL for the different spin-labelled constructs in **a**,**c**. (**e**) A cartoon representation of the electrostatic interaction of the Glu (E6 and E9) residues on subunits *i* with Lys (K97) residue of the second adjacent (*i*+2) subunit. (**f**) A cartoon representation of the electrostatic interaction of the Lys (K5) residue on subunits *i* with Glu (E108) residue of the adjacent (*i*+1) subunit and phosphate group of a POPE lipid molecule. The position of M94 has been indicated as a purple sphere with respect to these residues in **e**,**f**.

In order to provide unequivocal experimental support for the effects of N-terminal deletion seen in FE simulations, we used sequential deletion of the N-terminal helix in combination with site-directed spin-labelled EPR spectroscopy (Fig. 4).

First, all truncations produce loss of sensitivity phenotypes where expression of these constructs cannot rescue MJF465 *E. coli* cells from hypo-osmotic downshock (Fig. 4a)^40^. The most severe loss of sensitivity was seen with the Δ2–7 construct, with all constructs requiring considerably more force to gate when probed using patch-clamp electrophysiology (Fig. 4b and Supplementary Fig. 4)^41^,^42^. In the crystal structure of MscL, the N terminus of one subunit (*i*) comes within close proximity of TM2 of the second-next neighbouring channel subunit (*i*+2). Using the calculated mobility parameter at position M94-SL, as a surrogate for the mobility of TM2, we see a sharp increase in mobility when more than five residues were deleted from the N terminus. The spectra for the WT channel and the deletion mutants in the resting state are shown in Fig. 4c, and the associated mobility parameters (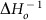) are quantified in Fig. 4d. We also show the spectra associated with the WT channel in the presence of LPC, which stabilizes the open state^16^,^43^. We can see that once we remove K5 the spectra of the deletions become progressively more like those encountered in the presence of LPC. This suggests that once K5 is removed the TM2 helix becomes more mobile and is consistent with the idea that an interaction between the N terminus and TM2 is lost. When we probed these interactions using MD simulations, we find that in fact a relatively strong electrostatic interaction is present between Glu residues on the N terminus (both E6 and E9) on subunit (*i*) and a Lys residue on TM2 of the second adjacent subunit (*i*+2; K97), which is ∼158.9±21 kcal mol^−1^ (Fig. 4e). The K5 residue in fact seems to interact in a molecular triad with a Glu residue that resides in the loops between TM2 and the C-terminal bundle on the adjacent subunit (E108) and the phosphate of the head group of a phosphatidylethanolamine (PE) molecule (Fig. 4f). This image again clearly shows that a lipid acyl chain protrudes into the inter-subunit cavity. This type of interaction with a phosphate group provides the necessary interaction between the channel and the bilayer, although not discriminating against the lipid type, an observation supported by the non-selective binding of lipids to MscL in Laganowsky *et al*.^44^.

### Structural rearrangements at the N terminus probed using EPR

**Figure 5:**
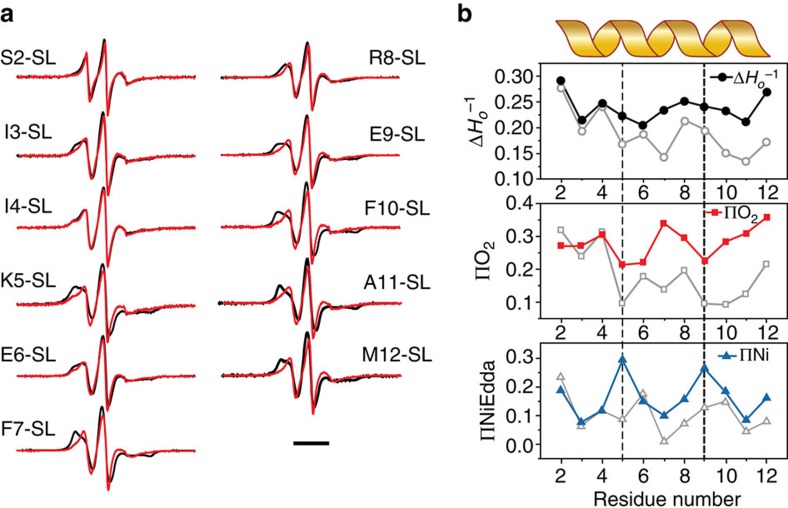
Conformational changes at the N terminus associated with the channel opening. (**a**) X-band CW-EPR spectra of liposome-reconstituted spin-labelled N-terminal mutants in the presence (red traces) and in the absence (black traces) of 25 mol% LPC to stabilize the open conformation. Experimental conditions as described in Fig. 3. Bar represents 30 G. (**b**) Environmental parameter profiles of the N terminus in the open state, derived from the spectra in **a** or from power saturation experiments. Mobility parameter Δ*H_o_*^−1^ (top panel, black circles), oxygen-accessibility parameter ΔO_2_ (centre panel, red squares) and NiEdda-accessibility parameter ΔNiEdda (bottom panel, blue triangles). For comparison, data for the closed state (from Fig. 2) are shown on each panel as grey open symbols. The vertical dashed lines point to the periodic maxima of NiEdda-accessibility and O_2_-accessibility minima in the open state.

Using LPC/PC mixtures, we proceeded to investigate the conformation of the N terminus as it transitions into the open state to complement our FE simulations. Figure 5a shows the spectra of each individual N-terminal domain residue (2–12) in the presence (Red) and absence (Black) of LPC. The difference in the mobility parameter and lipid and aqueous accessibilities are then quantified in Fig. 5b as discussed previously. Here the resting conformation is shown with open grey symbols and the filled symbols represent LPC-treated experiments. There is a large and periodic increase in NiEdda (aqueous) accessibility at positions 5 and 9. This ‘face' of the helix is opposite to the side with the large O_2_ (lipid) exposure, which includes residues 4, 7, 10 and 11. The full periodicity of the N-terminal region shown in the upper panel of Fig. 5b suggests that the N-terminal region is fully helical in the open state. Combined with the lipid and aqueous accessibility shown in Fig. 5b, we show that the N terminus is forming a single contiguous helix with TM1. In light of our structural model of open MscL we suggest that the transition into a fully helical conformation helps extend the now tilted TM1 to span the length of the bilayer^15^.

### Simulating the extension of the Gly14 linker

All the data so far support the integral role of the interfacial N-terminal helix as an essential force transducing element in the gating cycle of MscL. Given this importance, we wanted to further probe how the connection between the N-terminal domain and TM1 affects force transmission. In order to do this we extended the Gly14 linker that attaches it to the pore-lining TM1 helix. The idea here is that increasing the linker length will impair the transmission of mechanical force from the N terminus to the pore-lining helix. This was achieved by the insertion of extra glycine residues (either +2 or +5) in addition to the native Gly14 (Supplementary Fig. 5). Glycine was chosen because of its inherent flexibility and low helical propensity.

**Figure 6:**
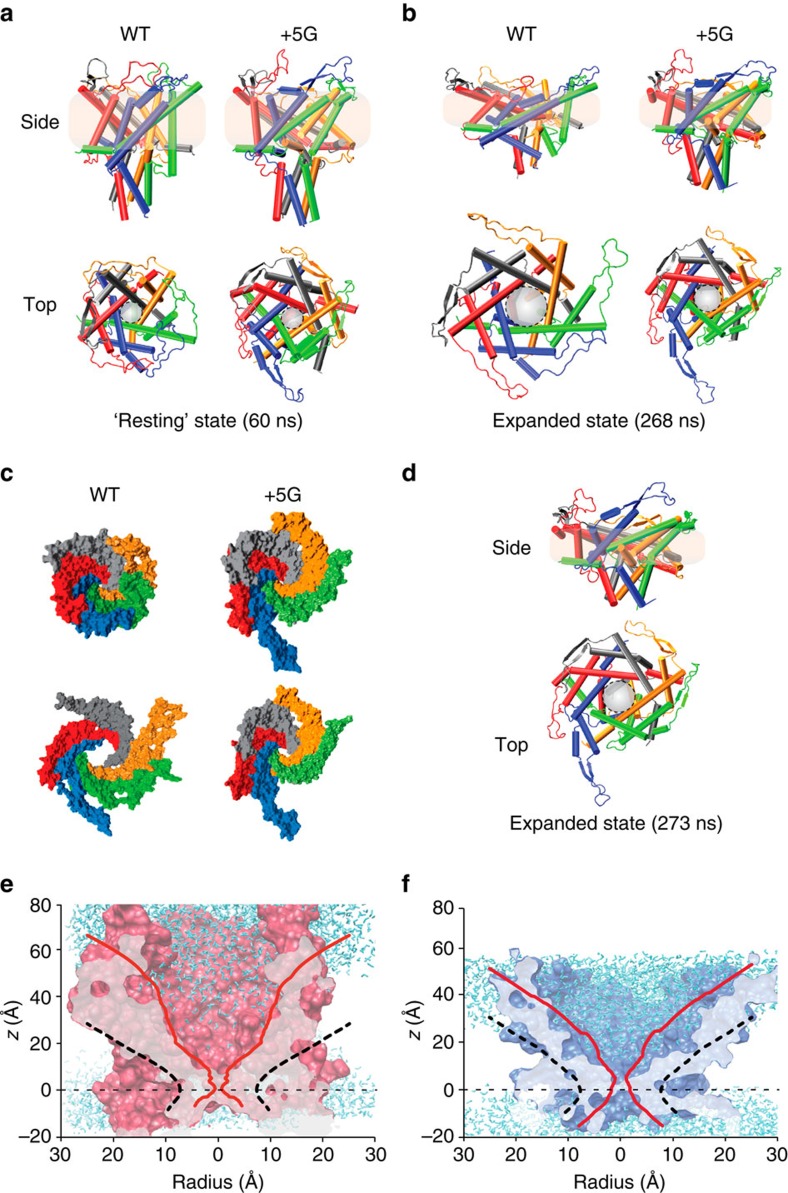
All-atom MD simulations of WT and +5G mutant EcMscL models. (**a**) Side and top views of WT MscL and +5G MscL in their resting equilibrated states. The TM1 helices in the +5G are slightly more tilted than in the WT model mostly because of the higher degree of freedom of TM1 in the +5G model. (**b**) Side and top views of WT and +5G MscL after 268 ns of simulation (for force regimen see Methods). The pore of the WT model is much more expanded than in the +5G model. This is because of the significant role of the N terminus in tilting TM1 in the membrane plane and in expanding the pore by driving the movement of TM1 away from the central axis. This ability is diminished by extending the Gly linker between the N terminus and TM1. (**c**) A surface representation of WT and +5G models in the resting and expanded states from the top. This clearly shows the difference in the degree of expansion. (**d**) The side and top views of the expanded state of the +5G mutant MscL model. To reach this state, the simulation was run for longer than the WT simulation (additional 5 ns under the high membrane surface tension of 100 mN m^−1^). (**e**) Closed (solid red) and expanded (dashed black) pore of WT MscL. (**f**) Closed (solid red) and expanded (dashed black) pore of +5G MscL. Comparing **e**,**f** shows that the upper regions of the +5G model in the closed state are substantially more expanded compared with WT because of an increased tilt of TM1 helices.

Our MD simulations were carried out using an optimized homology model of EcMscL based on the crystal structure of MtMscL and the C-terminal region of EcMscL from a recent crystal structure reported by the Rees group^17^,^18^. We then equilibrated a WT model and a model containing the additional five glycines (+5G) in a POPE (1-palmitoyl-2-oleoyl-sn-glycero-3-phosphoethanolamine) bilayer for 62 ns (Supplementary Fig. 6a,b). POPE was chosen as *E. coli* membranes contain >60% PE. The +5G mutant in the equilibrated closed state (after 62 ns) is more expanded, with an angle between the N-terminal helix and TM1 of 147±3° compared with 136±3° (mean±s.e.m.) in the WT channel (Fig. 6a–d). The s.e. is measured based on the difference observed among the angles in the five subunits over three repeated simulations (Supplementary Figs 5– 7). Consequently, the upper regions of the +5 G model in the closed state are substantially more expanded compared with WT because of the increased tilt of TM1 helices (Fig. 6c,d; repeats shown in Supplementary Figs 6 and 7). This also corresponds to a slight movement of the hydrophobic gate, which in the +5G mutant is centred around the V23 residue, whereas in the WT the gate is a composite of both L19 and V23 (Fig. 6 and Supplementary Fig. 7).

In order to see a partial gating transition over the time frame of our simulations (∼270 ns), it was necessary to apply higher tensional forces than those defined experimentally, taking into account that the first and midpoint activation tensions of MscL are 9 and 12 mNm^−1^, respectively^43^. We used 75 and 100 mNm^−1^ surface tension, but in an NγPzT ensemble^45^. This means when a surface tension of 75 mNm^−1^ is applied to a POPE bilayer, the bilayer is only stressed by ∼25 mNm^−1^ (Supplementary Fig. 6c). This is because each lipid bilayer has an intrinsic surface tension (for example, ∼50 mNm^−1^ for POPE) to keep its area per lipid constant when it is stress-free^46^, otherwise the bilayer would shrink^45^. Therefore, when we set the surface tension to 75 mNm^−1^, in fact we are increasing the surface tension of the bilayer by ∼25 mNm^−1^. In order to systematically show this, we have calculated the surface tension of the bilayer in the presence of MscL when it is stress-free and when it is stressed. Thus, the highest value we applied on our lipid bilayer is actually 3–5 × of the experimental value. Using higher membrane tension than the experimental range for MscL activation is a common issue with these types of MD simulations. However, it is currently a ‘necessary evil' in order to be able to capture full gating transitions, given existing limited computational timescales.

Our expanded structure obtained from MD simulations aligns well with the expanded structure in our FE model (Supplementary Table 1). This is a clear illustration that the FE model shows a similar trajectory to that of MD simulations, in particular, the formation of an almost contiguous helix (Fig. 6a–e and Fig. 3c) and is consistent with our extensive EPR data. It is important to note here that the addition of extra glycines does not affect the total lipid–protein interaction energy of our N-terminal helices, and it is instead a completely mechanistic insertion designed for testing our hypothesis (total interaction energy of the N terminus (the initial 13 residues) with the bilayer; WT 797.7±44 kcal mol^−1^, +5G mutant 841.3±63 kcal mol^−1^). Moreover, the interaction of the added five glycine residues with the lipid bilayer is in comparison negligible during our simulations (<1.6 kcal mol^−1^).

After 268 ns of MD simulation under identical conditions (for the exact force regimen see Methods), the level of upward tilting of TM1 is not as pronounced in the +5G mutant when compared with the WT (Fig. 6b, side view). In addition, the pore of the WT model is substantially more expanded than the pore in the +5G model (Fig. 6b,e). This is because of the significant role of the N-terminal domain in tilting TM1 in the membrane plane and in expanding the pore by driving the movement of TM1 away from the central axis of the pore. This ability is impaired by extending the Gly14 linker between the N terminus and TM1. To be able to expand the +5G pore to the same level as the WT pore, the same level of tension is needed for a further 5 ns (Fig. 6c–f).

Here we should also note that during the expansion of the WT model the tight association of the lipid molecules with the N-terminal helix is conserved (Fig. 2c–e). While these lipids are ‘dragged' away from protruding into the structure (Fig. 1c–e), their interactions with the protein, nevertheless, remain.

### Electrophysiological effects of extending the Gly14 linker

**Figure 7:**
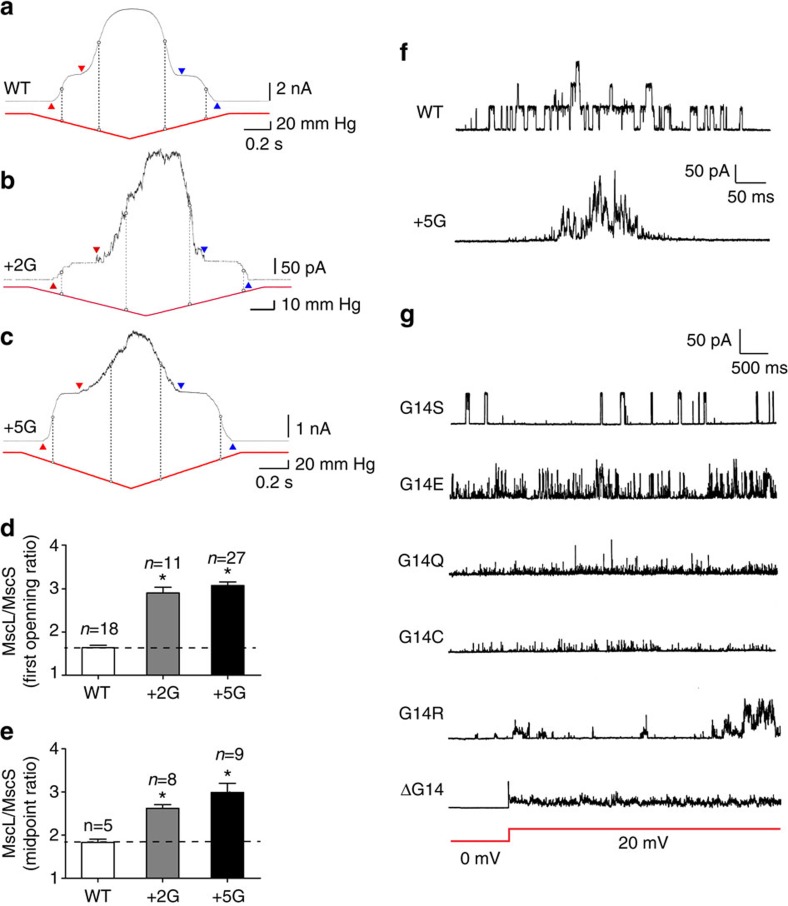
Effect of extension of the Gly linker between the N terminus and TM1 on MscL activation threshold in liposomes. MscS and MscL are co-reconstituted in 100% azolectin lipid. (**a**–**c**) Current traces of MscS and MscL recorded at +5-mV pipette potential. Red arrowheads point to the first observed MscS and MscL opening, used to determine the MscL/MscS first opening threshold ratio (TR). Blue arrowheads point to the last observed MscS closure and MscL closure. The vertical dashed line illustrates the midpoint activation threshold of the respective channels. (**d**,**e**) The comparison between activation TR of WT with +2G and +5G mutant MscL mutants. It can clearly be observed that extension of the G linker increases the activation threshold of MscL by almost 100%. (data points represent mean±s.e.m. *P-value<0.01; one-way ANOVA). (**f**) Single-channel recordings from WT MscL (upper panel: 3.04±0.04 nS (*n*=5)) and +5G MscL (lower panel: 2.76±0.08 nS (*n*=5)) both recorded at +20-mV pipette potential and after application of negative pressure. The +5G mutant channel gates at substates for a large percentage of the time especially when compared with WT, a fact largely because of the greater conformational freedom of the TM1 helix. (**g**) Effect of polar substitutions at position G14 on single-channel activity of MscL. G14S gives almost WT-like activity, whereas deletion of G14 results in a channel that gates spontaneously at substates, giving rise to a ‘leaky' toxic phenotype when expressed in *E. coli*. For ΔG14, the record shows the change in voltage from 0 to 20 mV pipette potential (red line) and the corresponding spontaneous activity.

In order to examine the functionality of mutant MscL channels with extended Gly14 linkers (+2G and +5G mutants), we purified the proteins and reconstituted them into azolectin liposomes (Fig. 7a–d). Owing to the complexity of electrophysiological patches and the impact of pipette geometry on membrane tension here we use MscS as a gauge to determine whether the extension of the linker between the N-terminal and the TM1 helix affects the MscL activation threshold^47^,^48^. When co-reconstituted with MscS, a severe loss of sensitivity to applied force was seen for both the +2G and +5G mutants (WT *P*_1/2_ ratio: 1.8±0.07 (*n*=6)+2G *P*_1/2_ ratio: 2.6±0.06 (*n*=8) and +5G *P*_1/2_ ratio: 3.0±0.21 (*n*=9)). In addition to these pronounced effects on opening, the lengthening of the G14 linker also mildly delayed closing (pressure-of-first-opening/pressure-of-last-closing ratio—WT: 1.0±0.03 (*n*=11), +5G 1.5±0.12 (*n*=19)) (Fig. 7d,e and Supplementary Fig. 9). However, the effect on closing was not as large as the effect on the mechanical force required to open the channel.

We saw the same loss of sensitivity in native membranes of *E. coli* spheroplasts (MJF612: MscL^−^, MscS^−^, MscK^−^ and YbdG^−^) when expressed with MscS (Supplementary Fig. 8). In fact, it was extremely difficult to apply sufficient force to gate the +5G mutant in spheroplasts and this is why we chose to characterize these channels in liposomes (Supplementary Fig. 9).

### The N terminus dictates the conformational freedom of TM1

One striking feature of extending the glycine linker between the N-terminal helix and the pore-lining TM1 helix is the change in single-channel activity. The +5G mutant gates almost exclusively in substates (Fig. 7f), making estimation of the channel unitary conductance difficult. This is indicative of the increased conformational freedom of TM1 enabled by the partial decoupling of the N-terminal helix. In the WT channel this tight link coordinates the movement of single monomers within the channel pentamer by efficiently coupling membrane tension to the TM1 helix of a single subunit.

Further mutational analysis of the Gly14 linker reveals its crucial role and the necessity for a relatively small hydrophilic residue in this region. Substitution with large residues prevented channel function completely (V, W), while the channel continued to function with polar side chains (S, Q, E, R; Fig. 7g). Deletion of G14 resulted in channels that were spontaneously active, giving rise to a ‘leaky' phenotype and retarded growth in *E. coli*. The activity represented gating in lower substates, and full channel openings could not be seen even with the application of high pressure to the patch pipette (Fig. 7g). This may well be because of a repositioning of the N-terminal helix as a result of a loss of the ‘kink' formed by G14. This is again further support for the stabilizing role of the N-terminal helix in the closed state of MscL.

## Discussion

Here we have fully investigated the role of the amphipathic N-terminal helix in the gating cycle of *E. coli* MscL. These types of helices are present in all sorts of membrane-associated proteins (not just membrane-spanning channels) and were first suggested by Segrest *et al*.^49^ to interact directly with the lipid bilayer. Mutagenic studies have previously shown the importance of this region in gating, particularly the phenylalanine residues^25^,^41^,^50^. Using an interwoven multidisciplinary approach combining experiment and simulation, we show that the short amphipathic N-terminal helix of MscL acts as a horizontal coupling helix linking membrane bilayer dynamics to protein conformation, as initially proposed by Iscla *et al*.^25^. Our simulations and EPR spectroscopy data suggest a firm interaction between the amphipathic N-terminal helices of MscL and the lipid bilayer, with numerous residues buried within the acyl chains (Figs 1 and 2 and Supplementary Fig. 10). This fits well with the work of Blount and colleagues, showing that mutating these buried hydrophobic residues, especially F7 and F10, results in channels with a higher activation threshold^25^,^51^. Thus, we postulate that the N-terminal helix (or its equivalent) is essential in the process of coupling bilayer forces to the pore-forming helices in a plethora of MS channels, a fact supported by robust experimental and computational data from numerous laboratories^11^,^27^,^28^,^29^,^30^,^31^,^52^.

**Figure 8:**
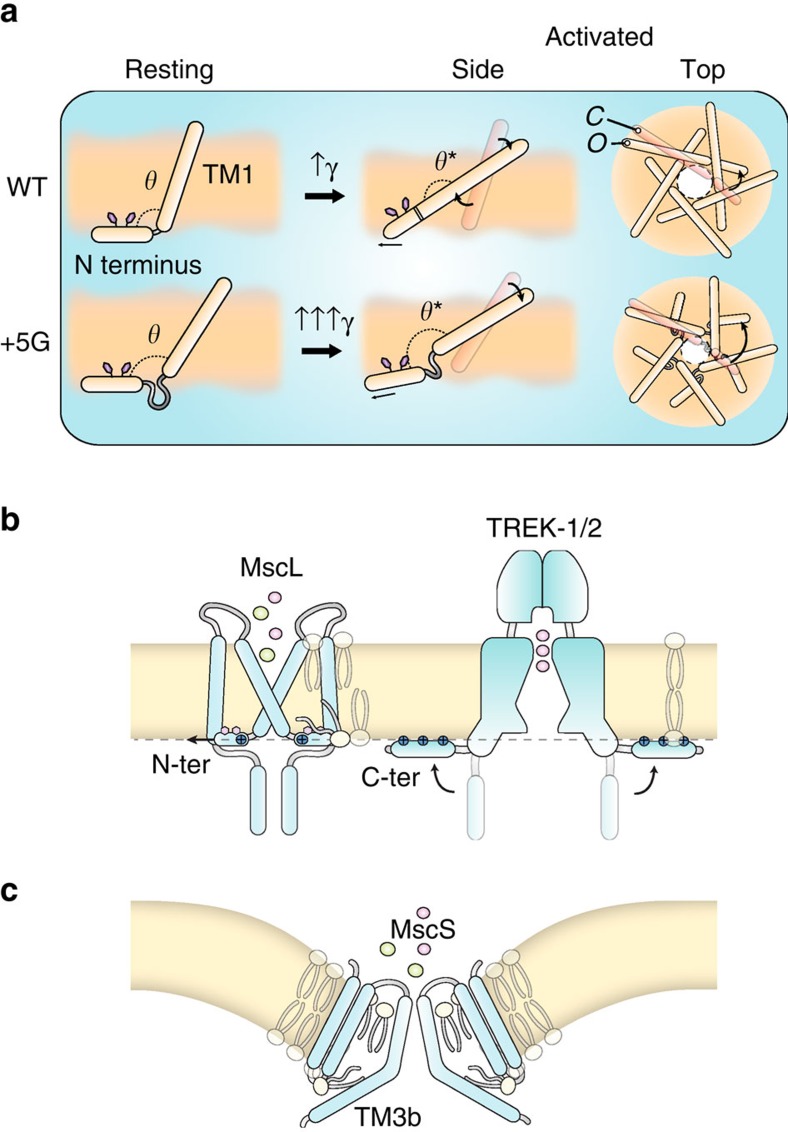
Crucial role of the N-terminal domain in the gating of MscL. (**a**) The N-terminal domain interacts tightly with the lipid bilayer. It orients at the lipid–solvent interface on the cytoplasmic side and acts as an anchor in stabilizing the closed state of the channel. As the lipid bilayer is stretched, the N-terminal helix is stretched following the trajectory of its surrounding lipid molecules but never becomes completely buried. Then, force is transmitted via the Gly14 residue to the end of TM1, which causes the alignment of TM1 with the N-terminal domain and the formation of a contiguous helix. If we extend the Gly linker (+5 G), we observe that one of the main roles of the N terminus is in expanding the pore. These functions are largely diminished in the +5G model because of the fact that TM1 does not follow the trajectory of the N-terminal region. (**b**,**c**) Horizontal membrane-coupling helices seem to be a hallmark of mechanosensitive channels. These helices maybe buried as in the N terminus of MscL, TM3b of MscS and the S4–S5 linker of TRPV4 or adsorbed on the membrane surface as in the C terminus of TREK channels. Owing to the various types of lipids present in different organisms and the divergent ways in which these coupling helices can interact, there is little to no necessity for sequence conservation despite the fact that they play an almost identical role.

Further, our MD simulations show that the acyl chains of the lipid molecules deform around the N terminus and protrude into an inter-subunit cavity, in agreement with recent observations in the bacterial channel MscS, and suggested for the two-pore domain potassium channel TRAAK (Fig. 8)^11^,^30^,^34^. We note that, while the acyl chains are removed from these cavities during gating, they still have a tight association with the N-terminal helix (Supplementary Fig. 11). Therefore, rather than a gating mechanism based on entropy-driven lipid acyl chain removal, MscL appears to open as a result of the direct ‘pulling' of structural elements in the channel such as the N-terminal helix. Thus, the ‘exclusion' of acyl chains from these cavities seems to be a consequence rather than a cause of MscL gating.

A critical finding in this study was that, during MscL activation, the N-terminal helix becomes a long, contiguous helix with TM1, as shown by EPR spectroscopy and FE/MD simulations (Figs 3 and 5). Overall, the results from FE and MD are very comparable as shown in the Supplementary Table 1. The overall stress map with high intensity in the N-terminal domain is consistent with the stress values determined by our and previous MD simulations (Supplementary Fig. 11)^53^. Complete deletion of the N terminus in our FE model resulted in a channel significantly less sensitive to applied membrane tension. This fact is clearly shown by the reduced pore expansion in our model that lacks the N terminus (Fig. 3e,f) and suggests that the N-terminal helix is an essential mechanosensing entity within the MscL structure. This is supported by electrophysiological data and hypo-osmotic downshock experiments, which demonstrated abrogated function of sequential N-terminal deletion constructs. EPR and MD simulations reveal that there are also essential interactions between the N terminus and the TM2 helix of the second subunit neighbour (*i*+2) in addition to the adjacent neighbour. These tight interactions define the conformational freedom of the TM2 helix (Fig. 4).

On the basis of the present data set and simulations, we suggest that the ‘force from lipids' is transmitted to the MscL pore-lining TM1 helices via the N terminus. This amphipathic helix would guide the tilting and movement of the five TM1 helices in a coordinated manner, magnifying the resulting pore expansion. Specifically, the conformational rearrangement that establishes the continuity between the N terminus and the intracellular end of the pore-lining TM1 helix allows a putative radial force on the N terminus to be transduced into increased tilt in the TM1 helix relative to the membrane. In this scenario, the link between the N terminus and TM1 (G14) is likely to play a critical role in mechanical coupling. Indeed, deletion of G14 leads to a ‘leaky' phenotype with continuous spontaneous activity at subconducting levels (Fig. 7g). This suggests that G14 likely acts as a hinge, positioning the N-terminal parallel to the membrane plane at the bilayer-solvent interface and that its structural flexibility is crucial for stabilizing TM1 (and thus, the closed state). This proposal is further supported by our site-directed mutagenesis at this position where less flexible residues (*W*, *V*) do not give rise to channel currents but more flexible polar residues do^54^, where a serine mutation gives rise to WT-like currents.

By averaging all the subunits, the N-terminal helix moves quite significantly in the radial direction during gating: 8.3±1.3 Å in MD simulations and 10.5±0.5 in FE simulations (Supplementary Table 1 and Supplementary Movie 1 and Supplementary Software 1). To further address the mechanism of force transmission from the N terminus to the TM1 helix, we designed mutants where the N-terminal/TM1 linker was extended by the addition of two or five glycines. In electrophysiology experiments, the linker extension resulted in channels with a much higher gating threshold, with these channels gating almost exclusively in substates because of the lack of coordinated movement of single subunits of the channel pentamer. A simple explanation for this is that the resulting increase in conformational freedom of the TM1 helix associated with this partial decoupling from the N terminus results in an abrogated ability of forces felt by the N terminus to be transferred to the TM1 helix. From MD simulations, the addition of five glycines should not affect the total interaction energy between the N termini and the bilayer. Our equilibrated +5G model displays a more expanded state, indicative of the partial decoupling of TM1 helices from the N termini, ultimately preventing the N-terminal stabilization of the closed state (Fig. 6). These results are in agreement with the recently published structural data on the role of the N-terminal domain in stabilizing the closed state of an archaeal MscL homologue MaMscL^55^. Therefore, in addition to the mechanosensing residues at the rim of MscL, for example, F78 (ref. 56), we conclusively show that the force transmission from the bilayer to the N terminus and thus to TM1 is an essential driver of both the tilting of TM1 and the radial expansion of the channel pore (Fig. 8a).

Horizontal membrane-coupling helices, such as the N-terminal domain of MscL, appear to be a hallmark of MS channels that gate according to the force from lipid paradigm and seems to be independent of their evolutionary provenance (Fig. 8b). These helices can be partly buried as we have shown here for the N terminus of MscL. This may also be the case for TM3b of MscS (it is important to note here that there are differing schools of thought regarding the exact gating cycle^34^,^57^) and the S4–S5 linker of TRPV4 (refs 31, 52). Alternatively, these mechanical-force-coupling helices may be adsorbed on the membrane surface through electrostatic interactions. Such regions are seen in the current crystal structures of TREK1/2 and TRAAK where the lower half of these channels seem to have a tight association with the lipid bilayer^11^,^12^,^30^,^58^. In addition, the distal C terminus in these force-sensitive K_2P_ channels, which is not seen in any of the TREK crystal structures, is predicted to be an extended amphipathic helix functionally essential for mechanically induced gating^27^,^28^,^29^. Regardless of the complexity of the gating paradigms for these individual classes of channels, these horizontal force-transmitting helices have repeatedly been shown to play integral roles in the transmission of mechanical stimuli according to the force from the lipid concept^12^,^15^,^33^,^59^,^60^. Owing to the various types of lipids present in different organisms and the divergent ways, these coupling helices can interact (buried, adsorbed); there is little to no expectation for sequence conservation, despite the fact that they play an almost identical mechanical role. Given that Piezo1 has recently been shown to be gated according to the force from the lipid paradigm^10^, we speculate, taking into account functional data^61^, that the α4^anchor^, which runs roughly parallel to the inner bilayer leaflet^13^, may also serve the same purpose in the gating of Piezo1 channels as the N terminus does for MscL.

## Methods

### Bioinformatics

Sequence alignments were carried out using Clustal Omega. Motifs were defined using Prosite. The following consensus motif was used to search UniProt for proteins containing a similar sequence: F-[K,R]-x-F-[A,I,L]-x-[K,R]-G. Helical wheel diagrams and hydrophobic moment (<μH>) were calculated using HeliQuest.

### FE model

Owing to large deformations during MscL gating, we employed an FE computational method implemented in the ABAQUS commercial software (Abaqus/Standard; Dassault Systems Simulia Dassault Systemes Simulia Corp., Providence, RI, USA). In our model, the α-helices are modelled as elastic rods and the loops are modelled as nonlinear wires. Elastic rods are fitted to the orientation of backbone carbons of each TM helix, while the loops are modelled as nonlinear wires. Although our FE model shares similarities with the previous FE models^36^,^38^,^62^, it differs from those in several important aspects as follows. Our model is based on a homology model of a more recent MscL crystal structure (PDB: 2OAR)^17^, showing that the N terminus connects the TM1 helix to the lipid bilayer almost perpendicularly to TM1. The C-terminal helices are not included as it has been shown that they have negligible effect on the gating of *Ec*MscL^41^,^63^. Glycine 14 (G14) between the N terminus and TM1 is modelled by a Hinge Connector available in ABAQUS, reflecting the flexible nature of this residue. To more accurately capture the lipid bilayer behaviour during gating, a mean field-like lateral pressure profile for the bilayer is considered in the tail and head regions (see ref. 47 for further details). This way, unlike the previous FE models^36^,^38^,^62^, our simulation does not require any assignment of mechanical properties for different areas of the lipid bilayer. Moreover, a nondimensional FE analysis is carried out, which is independent of the protein elasticity modulus. The applied tensional stress on the membrane as well as the stress outputs are also nondimensionalized using the overall Young's modulus of the protein. Pairwise interactions between the lipid and outer surface of the TM helices are defined by fitting a van der Waals (vdw) stress function^64^. The surface interaction stress can be computed as a function of the distance between two hydrophobic surfaces by adopting the following equation^47^.


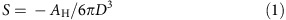


Here *S* is the stress, *A*_H_ is the Hamaker constant and *D* is the distance between the two surfaces. The normal tractions can be positive, indicating an attractive interaction between the surfaces, or negative, in the case of repulsive forces. The resulting expression of intralayer contact interactions has been implemented in an ABAQUS-user subroutine called UINTER for computing the vdw stresses. The lipid bilayer inner surface and the outer surface of each TM helix are defined as the master and slave surfaces, respectively. The UINTER subroutine is called for each individual slave node at every time increment through the analysis. Inputs to this subroutine are the initial and the incremental relative positions of each slave node with respect to its closest point on the master surface. The constitutive calculation thus involves computing the tractions based on the increments in relative position of the slave node with respect to the master surface. The energetic interaction constants are obtained from fitting equation (1) to the overall interaction energies obtained from our MD simulation of MscL in a POPE lipid bilayer. As a result, a Hamaker constant of ∼21(±3) × 10^−21^ J is obtained at the equilibrium state.

In the *Ec*MscL crystal structure, the TM1 and TM2 helices of adjacent subunits have a very large contact area stabilized by vdw interactions, and their crossing angle is slightly positive (∼10°). The contacts in the channel area involve the TM1 helices of the two adjacent subunits (crossing angle ∼–43°) and two TM2 helices: one within the same subunit (crossing angle ∼135°) and the second from an adjacent subunit (crossing angle ∼169°). The TM2 helices of neighbouring subunits do not directly contact each other, but are separated by ∼20 Å (refs 14, 17). N-terminal helices make an ∼95° link, with the TM1 close to the pore with the upper half of the N terminus buried in the lipid bilayer. Interhelical interactions are ignored for simplicity and more importantly to check the gating characteristics of MscL when only force transmitted from the membrane is considered. Before any FE analysis, a mesh-sensitivity study was carried out to ensure the independence of the results from the computational mesh. All the FE computational analyses were conducted using a CPU 2.4 GHZ, 8-Gigabyte RAM and at the maximum run time of ∼5 h.

### MD simulations

All MD simulations were performed with NAMD2.10 (ref. 65). Visual MD (VMD)^66^, Pymol and UCSF Chimera were used for all visualizations. The three-dimensional structure of *E. coli* MscL and +5G mutant EcMscL were generated based on the crystal structure of the MscL homologue of *M. tuberculosis* (PDB ID: 2OAR) using Phyre^2^,^67^ and Swiss-Model^68^.

The resultant MscL models were embedded into a POPE bilayer comprising 222 lipid molecules using the VMD software. After deletion of lipids inside the channel and those in very close proximity to the protein (<5 Å), the lipid heads and tails were in turn randomized and equilibrated for ∼1 ns at 298 K, while the rest of the system was fixed. The protein and lipids were next solvated with a 120 × 120 × 130–Å water box and 200 mM KCl. The TIP3P water molecule model was used along with the SOLVATE programme. A further randomization of the POPE lipid tails was performed, with the rest of system being fixed. Lipid, water and ions packed around the protein for 1 ns with the Cα atoms of the protein were restrained with a spring constant of 5 KJ mol^−1 ^Å^−2^ at constant pressure (1 atm). Then, the whole system was equilibrated for 62 ns with a time step of 2 fs with no restraints. The equilibration step (62 ns) was run three times for each of the WT and the +5G mutant models. After equilibration to see the channel expansion, a surface tension of 75 mN m^−1^ was applied on the lipid molecules for 200 ns with a time step of 1 fs in an NγP_z_T (constant surface tension, constant pressure and constant temperature) ensemble (262 ns total simulation time). To see a partial gating event of the WT and +5G mutated channels, a further 25 mNm^−1^ was added to the previous surface tension (100 mNm^−1^ in total) and applied to the membrane for another 6 ns (268 ns in total) and 11 ns (273 ns in total), respectively (1 fs time step). In all the simulations, the particle-mesh Ewald method was used for computing electrostatic interactions beyond a real-space cutoff of 1.2 nm with a Fourier grid spacing of 0.1 nm. A modified Nosé–Hoover Langevin piston pressure control provided in NAMD was utilized to control fluctuations in the barostat (at 1 atm). This method is combined with a method of temperature control (at 298 K; Langevin dynamics) in order to simulate the NPT ensemble. The CHARMM c36 Force field was used for all MD calculations. The pore shape of closed and open MscL channels was interrogated using the HOLE programme with a probe of 1 Å radius using the CHARMM36 vdw radii for the protein and energetic calculations done using the NAMD energy plugin.

We wrote custom tcl codes (Supplementary Software 1) for calculating the interaction energies, thickness, tilt angle and movement of the N-terminal helix. All custom-written codes are available for download.

### Mutagenesis and construct generation

In order to probe the role of the N-terminal helix, we created constructs that expressed MscL with a hexahistidine fusion purification tag specifically on the C terminus to prevent the tag from interfering with N-terminal gating mechanisms that were to be analysed by electrophysiology. MscL was subcloned into pETDuet-1 vector (Novagen) by PCR using PfuUltra (Agilent) thermostable DNA polymerase. The PCR primers (IDT; ‘BsaI-MscL-F' acttaaGGTCTCcCATGCGCGGGAACGTGGTGGATTT and ‘HindIII-MscL-6his-stop' tagctaAAGCTTTTAGTGGTGGTGGTGGTGGTGAGAGCGGTTATTCTGCTCTT) were used to amplify the WT *E. coli* MscL-coding sequence for the ligation into the NcoI and HindIII sites of pETDuet-1 after the PCR product had been digested with BsaI and HindIII-HF restriction enzymes (New England Biolabs). The reverse primer coded to the last WT amino acid, immediately followed by hexahistidine purification fusion tag and stop codon, while the forward primer allowed translation to start with the native start methionine. The construct allows MscL expression by T7 RNA polymerase-based *E. coli* extract cell-free synthesis systems (Exiprogen, Bioneer) or by *E. coli* recombinant culture systems compatible with the T7 RNA polymerase promoter.

Two extra glycines were inserted into the N-terminal MscL domain of the above construct immediately before G14 using a ‘Quikchange' site-directed mutagenesis method employing the following primers: ‘MscL R13R+GG-S' GAATTTCGCGAATTTGCGATGCGCGGTGGTGGGAACGTGGTGGATTTGGCGGTG and ‘MscL R13R+GG-AS' CACCGCCAAATCCACCACGTTCCCACCACCGCGCATCGCAAATTCGCGAAATTC.

The construct to encode for five extra glycines adjacent to G14 was constructed by performing megaprimer site-directed mutagenesis to the above ‘+2G' construct. The primary megaprimer PCR reaction used the following primer ‘MscL R13R+GGGGG-S' GAATTTCGCGAATTTGCGATGCGCGGTGGTGGTGGTGGTGGGAACGTGGTGGATTTGGCGGTG (to insert three more glycines ahead of the two extra glycines), with the ‘HindIII-MscL-6his-stop' primer. The secondary megaprimer PCR reaction used the resulting primary PCR product as a reverse primer with the ‘BsaI-MscL-F' primer to create a full-length +5G MscL C-terminal hexahistidine insert to be cloned as above into pETDuet-1.

### Protein purification and incorporation into liposomes

MscS and MscL were purified using immobilized metal affinity chromatography and either 6 × - or 10 × -His-tag combined with size exclusion chromatography^69^. Final elution buffers contained 1-mM n-Dodecyl β-D-maltoside for both channel proteins. Reconstitution into soybean azolectin (Sigma, CA) liposomes was carried out using the dehydration/rehydration (D/R) method^69^,^70^. Briefly, mixed lipids were dissolved in chloroform and dried under N_2_ flow to make a thin lipid film. D/R buffer (200 mM KCl, 5 mM HEPES, adjusted to pH 7.2 with KOH) was added before vortexing and in subsequent 10 min of sonication. MscS and MscL were added at a protein-to-lipid ratio of 1:500 (w/w) and incubated at 4 °C for 1 h. Detergent was removed with the addition of Biobeads (Bio-Rad, Hercules, CA) and incubated at 4 °C for further 3 h. The proteoliposomes were collected by ultracentrifugation and resuspended in 30 μl D/R buffer. Aliquots of proteoliposomes were spotted on cover slips and dehydrated overnight under vacuum conditions at 4 °C. The dried proteoliposomes were then rehydrated with D/R buffer at 4 °C for at least 6 h and subsequently used for electrophysiological experimentation.

### Spheroplast preparation

*E. coli* spheroplasts were prepared as using a standardized protocol^43^,^70^. Briefly, MJF465 (Δ*kefA*::kan,Δ*yggB*,Δ*mscL*::Cm) or MJF612 (*Δybdg*::apr,Δ*kefA*::kan,Δ*yggB*,Δ*mscL*::Cm) cells were grown for 1.5 h in the presence of the cephalosporin-type antibiotic cephalexin (final concentration 60 μg ml^−1^) to form *E. coli* snakes (length − 50 to 150 μm). Cells were then digested for 3–7 min in the presence of 0.8 M sucrose, 60 mM TRIS, pH 7.2, lysozyme (final concentration 0.2 mg ml^−1^) and EDTA (6.3 mM). A stop solution (0.8 M sucrose, 20 mM MgCl_2_, 60 mM TRIS pH 7.2) was added, and the spheroplasts were collected by centrifugation.

### Electrophysiology

An aliquot of proteoliposomes (1–3 μl) was introduced into the recording chamber. Channel activity of WT MscL and mutant proteins (+2G and +5G) were co-reconstituted with the WT MscS channel and examined in inside-out azolectin liposome patches using the patch-clamp technique^43^. Borosilicate glass pipettes (Drammond Scientific Co, Broomall, PA) were pulled using a micropipette puller (PP-83; Narishige, Tokyo, Japan). Pipettes with resistance of 3.0–5.0 MΩ were used for the patch-clamp experiments. Pipette and bath solution contained 200 mM KCl, 40 mM MgCl_2_ and 5 mM HEPES (pH 7.2 adjusted with KOH). In the case of spheroplast patching, the bath solution was supplemented with 400 mM sucrose. The current was amplified with an Axopatch 200B amplifier (Molecular Devices, Sunnyvale, CA), filtered at 5 kHz and data acquired at 20 kHz with a Digidata 1440A interface using pCLAMP 10 acquisition software (Molecular Devices) and stored in a personal computer. Negative pressure was applied manually with a syringe or High-Speed Pressure Clamp-1 apparatus (HSPC-1; ALA Scientific Instruments, Farmingdale, NY) and was monitored with a pressure gauge (PM 015R, World Precision Instruments, Sarasota, FL).

### Survivability of MJF465 cells

Survival of MJF465 (Δ*kefA*::kan,Δ*yggB*,Δ*mscL*::Cm) *E. coli* expressing different N-terminal deletion constructs of EcMscL after osmotic downshock from LB supplemented with 500 mM NaCl (∼1,000 mOsm) to LB was carried out using a standardized protocol^40^. Briefly, cells were exposed to osmotic downshock or control conditions for 15 mins, and 10 μl of a fivefold dilution was aliquoted on an agar plate. Plates were incubated overnight and the resulting number of colonies was counted the next day.

### Spin-labelling and EPR spectroscopy

The purified MscL mutant proteins were spin-labelled overnight with methanethiosulfonate spin label (Toronto Research) at a 10:1 label/channel molar ratio and were reconstituted at a 500:1 lipid/channel molar ratio by dilution in PBS^16^. Consequently, in a fully labelled channel, each of the five subunits had one spin label attached. EPR spectroscopy was performed using X-band CW EPR spectra obtained in a Bruker EMX spectrometer fitted with a loop–gap resonator at 2-mW incident power, 100 kHz modulation frequency and 1 G modulation amplitude^16^. The accessibility parameter used in this study to quantify the extent of solvent accessibility in a per-residue basis reports on the individual collision frequency of a nitroxide spin label with paramagnetic test compounds, and it is derived from the midpoints of signal saturation at increasing microwave powers. Power saturation curves were obtained for each spin-labelled mutant after equilibration in N_2_ as control, and air (21% O_2_), and N_2_ in the presence of 200 mM NiEdda as relaxing agents. All EPR data were obtained at room temperature.

### Statistical analysis

One-way analysis of variance combined with a Holm–Šídák *post hoc* test (Fig. 7 and Supplementary Fig. 8) or Student's *t*-test (Supplementary Fig. 9) were used for statistical analysis and differences were considered significant at *P*<0.01. Statistical significance was confirmed in groups with low *n* number using non-parametric Kruskal–Wallis test. The number of experiments and replicates is mentioned in the figure legends. The values are mean±s.e.m., unless otherwise specified.

### Data availability

The authors confirm that the data that support the findings of this study are available from the corresponding author upon reasonable request.

## Additional information

**How to cite this article:** Bavi, N. *et al*. The role of MscL amphipathic N terminus indicates a blueprint for bilayer-mediated gating of mechanosensitive channels. *Nat. Commun.* 7:11984 doi: 10.1038/ncomms11984 (2016).

## Supplementary Material

Supplementary Information Supplementary Figures 1-11 and Supplementary Table 1.

Supplementary Movie 1 Illustration of how the N-terminal helices (shown in red) move during the gating of MscL (top view). As reported in Supplementary Table 1, each N-terminal helix is pulled around 8 Å away from the central pore axis during the 205 ns of active simulation. For exact calculations of the distance N terminus moved during simulations see Supplementary Table 1.

Supplementary Data 1 Custom tcl codes for calculating the interaction energies, bilayer thickness, helix tilt angle and movement of the N-terminal helix. All custom written codes are available for download.
