# Supplementary material for: The role of MscL amphipathic N terminus indicates a blueprint for bilayer-mediated gating of mechanosensitive channels
Source: Nat Commun. 2016 Jun 22;7:11984. doi: 10.1038/ncomms11984 (PMC4917966; doi:10.1038/ncomms11984)
Supplement: Supplementary Information — Supplementary Figures 1-11 and Supplementary Table 1. [file ncomms11984-s1.pdf]

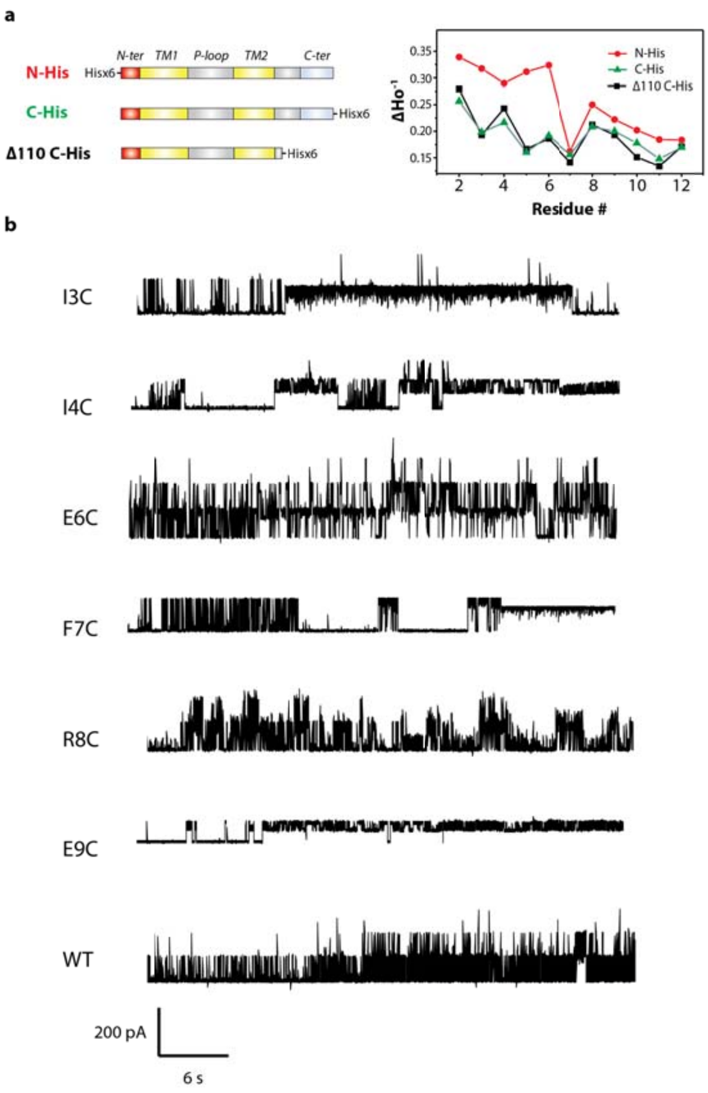

Supplementary Figure 1 The influence of the N-terminal tag on structure and dynamics of EcMscL. (**a**) Schematic diagrams of the three types of EcMscL constructs used in the present study (left panel) and local mobility profile for the three MscL constructs, as derived from line-shape broadening of spin-labeled mutants (right panel). (**b**) Representative current traces of N-terminal cysteine mutant channels. Except for the mutant K5C all other mutants were functional at the single channel level. Most of them displayed frequent and/or prolonged gating at subconducting levels.

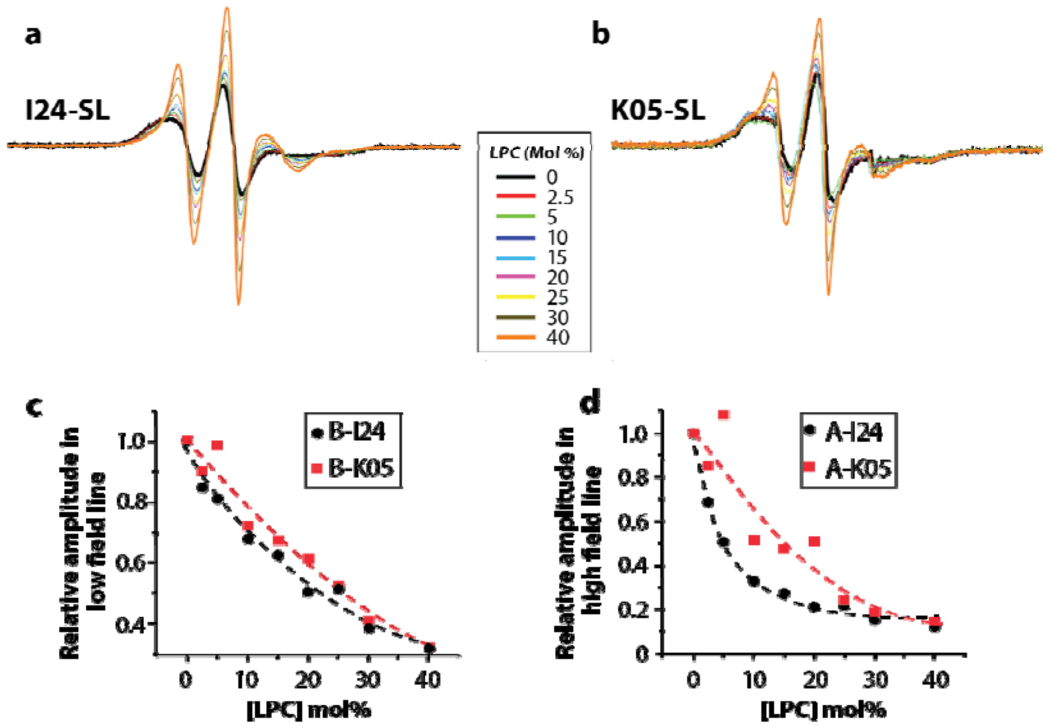

Supplementary Figure 2 Comparison of the loss of function phenotype of K5C with a WT-like mutation in the TM1 helix (I24C). (**a**) X-band CW-EPR spectra of spin-labeled I24C and (**b**) and K5C mutants reconstituted into azolectin liposomes. Spectra are color coded according to the concentration of the LPC used for the MscL activation (middle) (**c,d**) Low and high field response to different concentrations of LPC of K5C and I24C mutants. The figures show that more LPC is required to gate the K5C mutant but nevertheless it can ultimately adopt the fully open state.

a

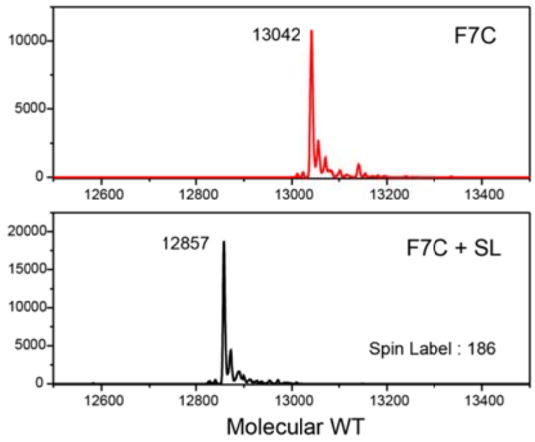

b

Residue #	% M TS-S L Labelin g
S2C	> 99 %
I3C	> 99 %
I4C	> 99 %
K5C	~ 91 %
E6C	> 99 %
F7C	> 99 %
R8C	> 99 %
E9C	> 99 %
F10C	> 99 %
A11C	> 99 %
M12C	> 99 %

Supplementary Figure 3 Mass spectrometry of the spin labeling efficiency. The extent of labeling of the N-terminal residues was directly determined from their mass spectra by integrating the respective peaks for the expected masses of the labeled and unlabeled (**a**) species using the electrospray ionization technique (31). All N-terminal residues examined by EPR spectroscopy were fully labelled (**b**).

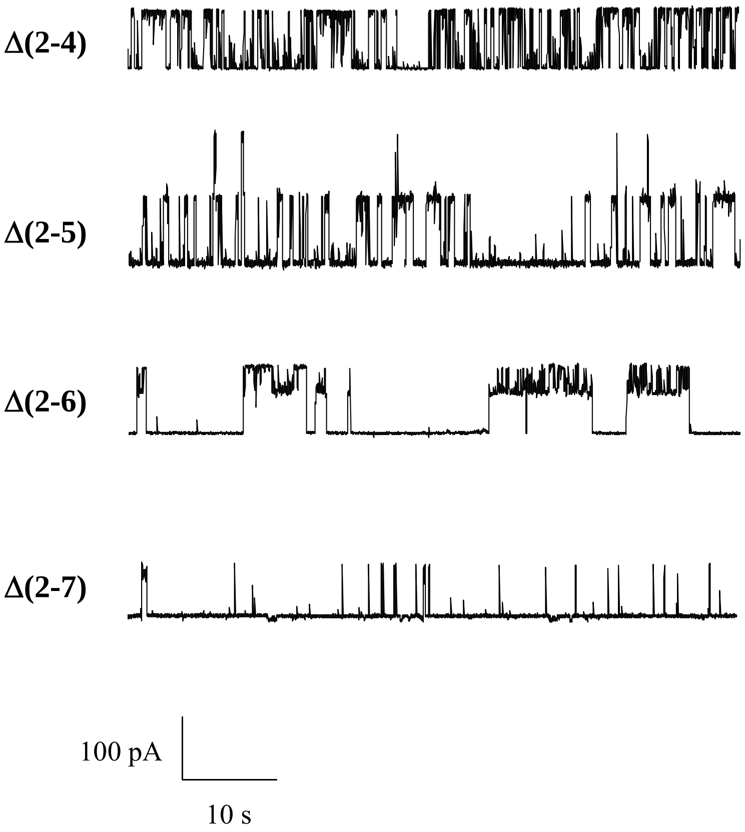

Supplementary Figure 4 Current traces of N-terminal deletion mutants. The activity of the deletion mutants decreased as the size of the deletion was increased. All mutant channels were reconstituted into azolectin liposomes and recorded at a pipette voltage V_p = +30 mV and suction (negative pressure applied to the patch pipette) of 60 mmHg.

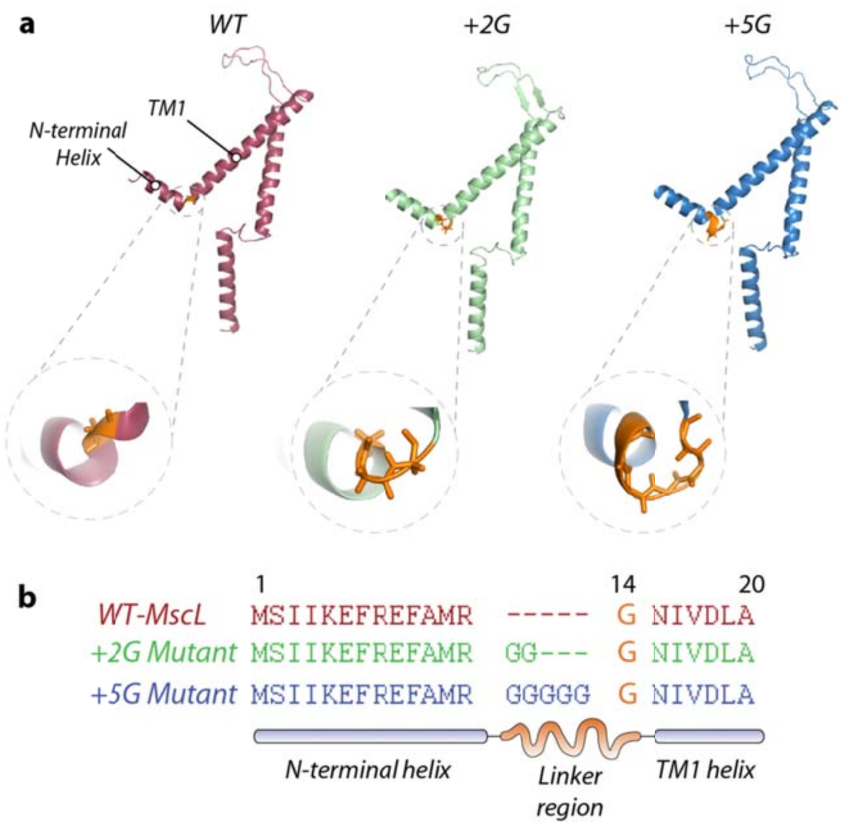

Supplementary Figure 5 Extension of the glycine linker between the N-terminal helix and TM1 helix. (**a**) In addition to the wild type (WT) model (red), two *in silico* models of +2G (green) and +5G mutants (blue) are shown, where two and five extra glycine residues are added to G14, respectively. (**b**) The sequence of the initial amino acids of WT, +2G and +5G mutant models showing the location of the extra glycine residues.

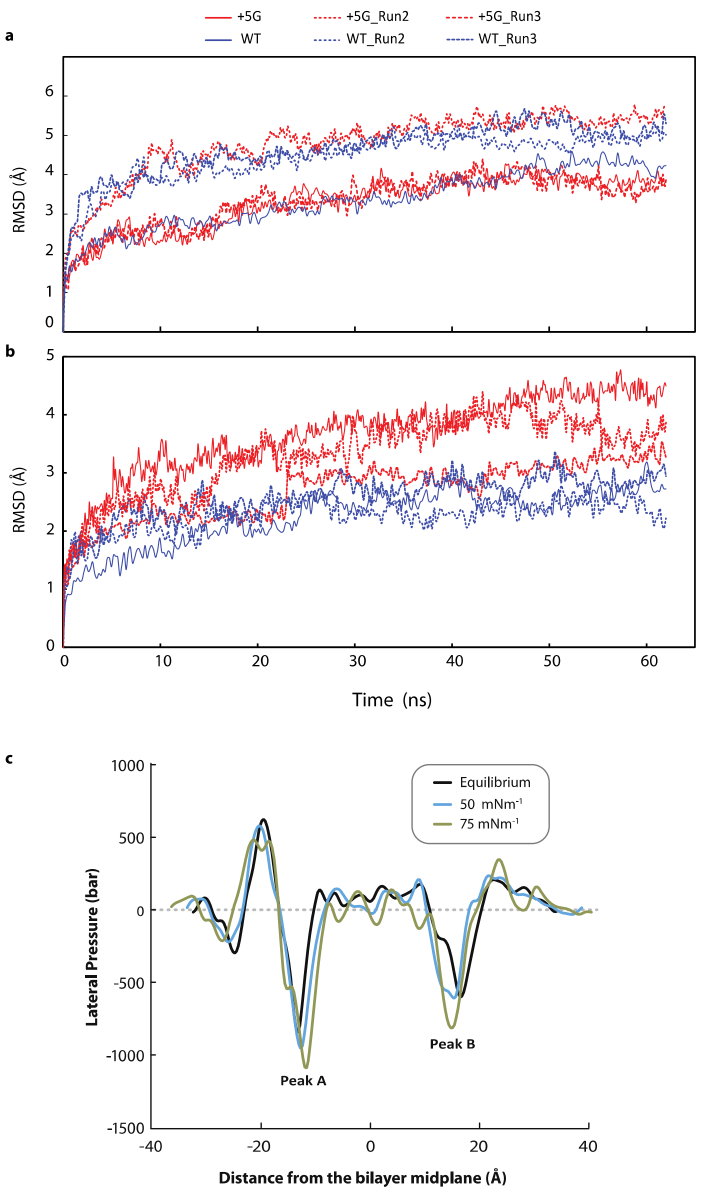

Supplementary Figure 6 Equilibration of WT MscL and +5G mutant in a POPE lipid bilayer in an NPT ensemble using all-atom MD simulation. Simulation for each model (WT and +5G) was carried out three times, each for 62 ns duration. (**a**) Root Mean Square Deviation (RMSD) of the protein backbone carbons plotted against simulation time. Both WT and +5G models are well equilibrated after 62 ns. (**b**) During equilibration, the RMSD of N-terminal domain in WT is lower than the RMSD of +5G N-terminal region as the latter one has more freedom due to the extra added glycines. (**c**) Illustration of how the pressure profile of the lipid bilayer in the presence of MscL changes at different surface tensions of 50 and 75 mNm^{-1} in an NγPzT ensemble. Also of note is the asymmetry in the pressure profile after MscL has been inserted into the bilayer. As shown, when the target surface tension is $50mNm^{-1}$ the pressure profile of the lipid in the presence of MscL changes negligibly. We only start to see noticeable changes in the pressure profile at 75 mNm^{-1}, particularly at the

lipid water interface (Peak A and Peak B). In this condition (target surface tension of 75 mNm^{-1}), compared to a non-stressed bilayer (equilibrium) and based on the area under the pressure profile we estimate a mean membrane tension of 25.5 mNm^{-1} has been applied on the membrane, which is about twice the experimental value.

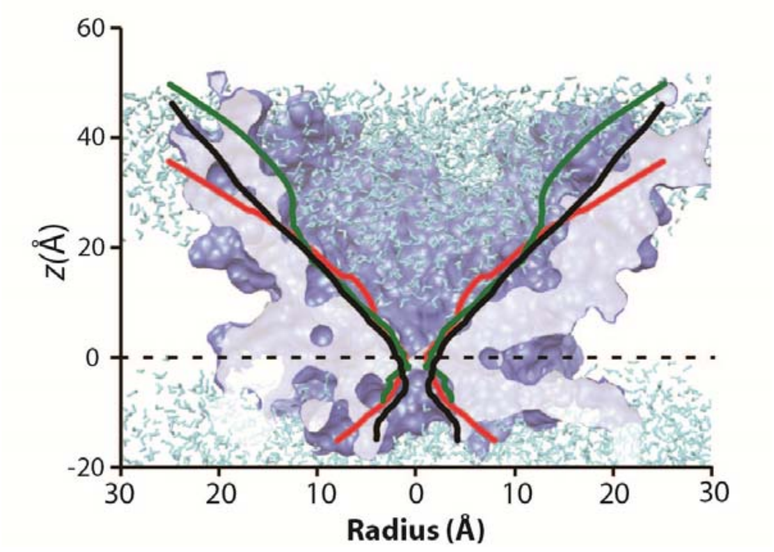

Supplementary Figure 7 Pore shape of +5G mutant MscL equilibrated in a solvated POPE lipid bilayer for 62 ns. The simulation is run three times and the pore shape has been shown for each run. This clearly supports the idea that as the Gly linker between the N-terminus and TM1 helices gets longer, the resting conformation of the channel becomes wider compared to the WT model. The red, green and black lines represent the reproducibility of this expansion in our 1st, 2nd and 3rd simulations respectively. Moreover, the length of the hydrophobic constriction region of the pore is shorter than in the WT MscL largely due to higher degrees of freedom of the N-terminus in the +5G mutant MscL.

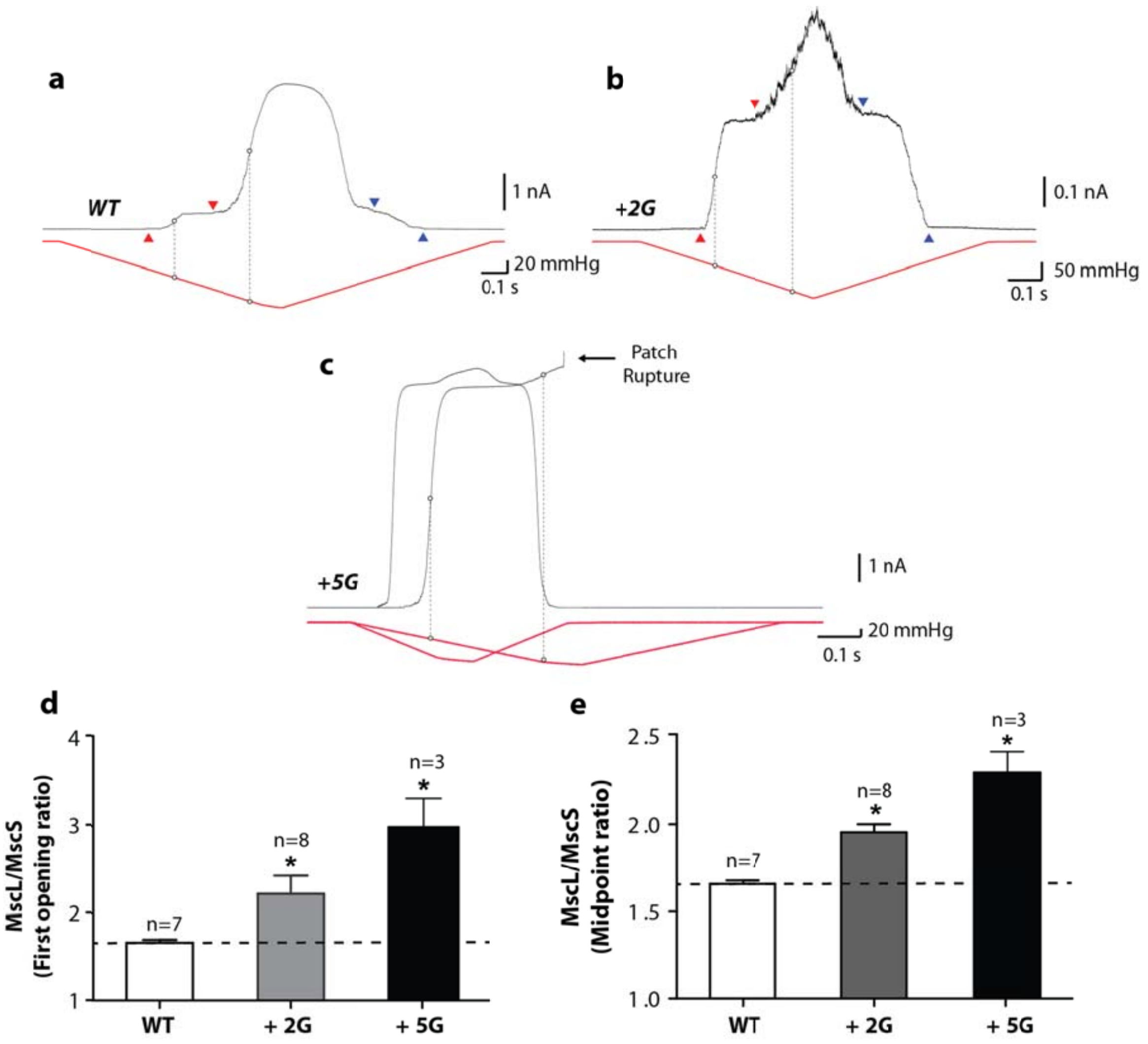

Supplementary Figure 8 Effect of extension of the Gly linker between the N-terminus and TM1 on MscL activation threshold in *E. coli* spheroplasts. (**a-c**) Current traces of MscS and MscL recorded at +10 mV (pipette voltage). Upward red arrowheads point to the first observed MscS opening, whereas the downward pointing arrows indicate the first observed MscL opening used to determine the MscL/MscS first opening threshold ratio (TR). The vertical dashed line illustrates the midpoint activation threshold of each channel [(**a**) WT MscL, (**b**) +2G and (**c**) +5G mutant MscL]. (**d**, **e**) Shows the comparison between activation TR of WT with +2G and +5G mutant MscL channels. It can clearly be seen that extension of the Gly linker greatly increases the activation threshold of MscL. Significant differences are indicated by asterisks (*p-value < 0.01; One-way ANOVA). [WT MscL/MscS $P_{1/2}$ 1.7 ± 0.03 (n=7) and first opening 1.7 ± 0.2 (n=7). +2G mutant/MscS $P_{1/2}$ ratio: 2.1 ± 0.06 (n=8), first opening ratio: 2.3±0.15 (n=8) and +5G mutant/MscS $P_{1/2}$ ratio: 2.3 ± 0.20 (n=3), first opening 3.01 ± 0.33 (n=3)]. In addition, the mutants also showed a decreased sensitivity to applied force indicated by an increase in 1/α values i.e. a shallower slope of the Boltzmann fit to P_o vs pressure [WT: 5.1 ± 0.6 mmHg (n=3), +2G: 8.7 ± 2.3 mmHg (n=3) and +5G: 12.3 ± 1.4

mmHg (n=3)]; where P_o is the open probability, and α is the slope of the plot ln $[P_o/(1-P_o)]$ vs $(p-p_{1/2})$.

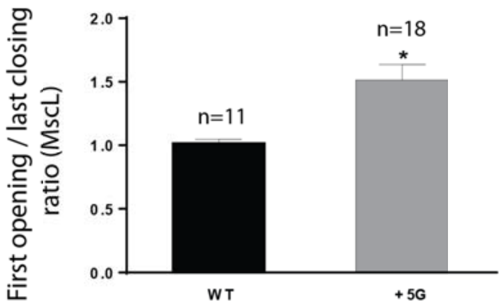

Supplementary Figure 9 First opening over last closing pressure threshold ratio of MscL. Full closing of +5G occurs at lower pressures compared to the WT. This is due to the fact that the reorganisation and closing of the pore by the N-terminal domain and TM1 helix is less effective in +5G mutant as a result of the extended Gly linker (Data represents mean ± S.E.M; p-value <0.01, Student's T-test).

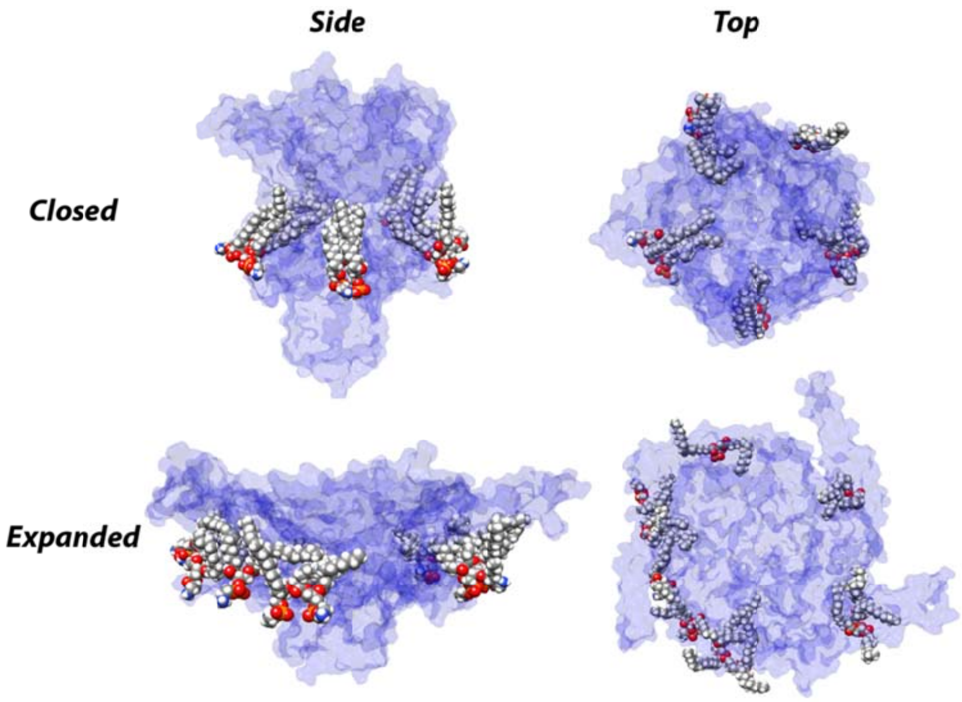

Supplementary Figure 10. Lipid protrusion in the inter-subunit cavities in the closed (resting) state (upper panel) and the open state of the channel (lower panel). From the side and the top views it is clear that the acyl chain of the lipid molecules protrude into the inter-subunit cavities in the resting state of MscL such that they interact tightly with the upper surface of the N-terminus. In the open state the lipid chains are stretched out of the cavities while they drag (guide) the N-terminus with them via their tight coupling.

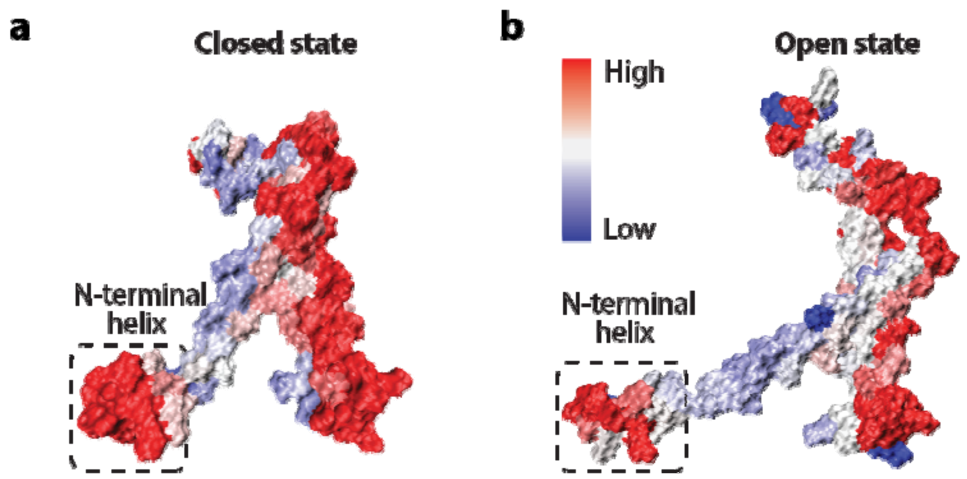

Supplementary Figure 11 Lipid-protein interaction mapped onto an EcMscL subunit from MD simulations. The interaction energy is shown between the lipid and the protein in (**a**) the closed state and in (**b**) the open state. Red shows the highest interactions and blue shows the lowest interaction. This unsurprisingly illustrates the highest level of interaction between the lipid facing part of TM2 and the N-terminal helix. (**a**,**b**) Illustrates that the N-terminal has an intimate interaction with the lipid bilayer in the closed state and that this tight association is conserved throughout the gating cycle. This is commensurate with the idea that the N-terminal is being 'pulled' by the lipid and drives gating.

		MD			FE	
		Initial angle	Final angle	Tilt angle	Final angle	Tilt angle
**Tilt about Z axis**	N-terminal	76.3 ±6.2	87.3 ±5.7	12.0 ±10.2	88.6 ±1.2	13.9 ±1.2
	TM1	47.0 ±1.0	70.8 ±1.3	23.8 ±1.7	72.1 ±2.9	25.0 ±2.7
	TM2	30.6 ±2.2	60.5 ±3.1	30.0 ±3.5	52.9 ±4.6	26.3 ±2.4
	Pore Diameter (Å)		Bilayer thinning (%)		N-terminal movement (Å)	
**MD**	28.2		15.3		8.3 ±1.3	
**FE**	30.1		14.3		10.5 ± 0.5	

Supplementary Table 1 Comparison of the MscL structure obtained from all-atom molecular dynamics (MD) and finite element (FE) simulation in the closed (initial) and the open state (final). The initial angles of FE are exactly the same as in the MD since it was mapped from the MD structure. All the angles are in degrees. Z axis is the direction normal to membrane plane (along the membrane thickness). It is notable that the MscL pore obtained from MD simulation is not completely round in shape (**Fig. 2C**) with a maximum pore diameter of 28.2 Å. The pore shape obtained from FE is more symmetric and the diameter is ~ 30.1 Å. Since the FE structure is modelled based on the backbone carbons (no side chains), the reported values for the pore diameter for both MD and FE simulations are based on the backbone to backbone distance of the constriction pore which structurally encompasses L19 and V23. All the values are Mean ± SEM and n=5. The N-terminal helix movement obtained from the MD simulation is 8.3 ±1.3 Å versus 10.5 ± 0.5 Å obtained from the FE simulation. The bilayer thins 14.3 % after full MscL gating in our FE model. The thickness change due to MscL gating obtained from MD simulation is 15.3 %.
